# Probing the Distribution and Mobility of Aminopolymers after Multiple Sorption-Regeneration Cycles: Neutron Scattering Studies

**DOI:** 10.1021/acs.iecr.4c01595

**Published:** 2024-08-14

**Authors:** Hyun June Moon, William T. Heller, Naresh C. Osti, MinGyu Song, Laura Proaño, Ida Vaghefi, Christopher W. Jones

**Affiliations:** † School of Chemical & Biomolecular Engineering, Georgia Institute of Technology, Atlanta, Georgia 30332, United States; ‡ Neutron Scattering Division, Oak Ridge National Laboratory, Oak Ridge, Tennessee 37831, United States

## Abstract

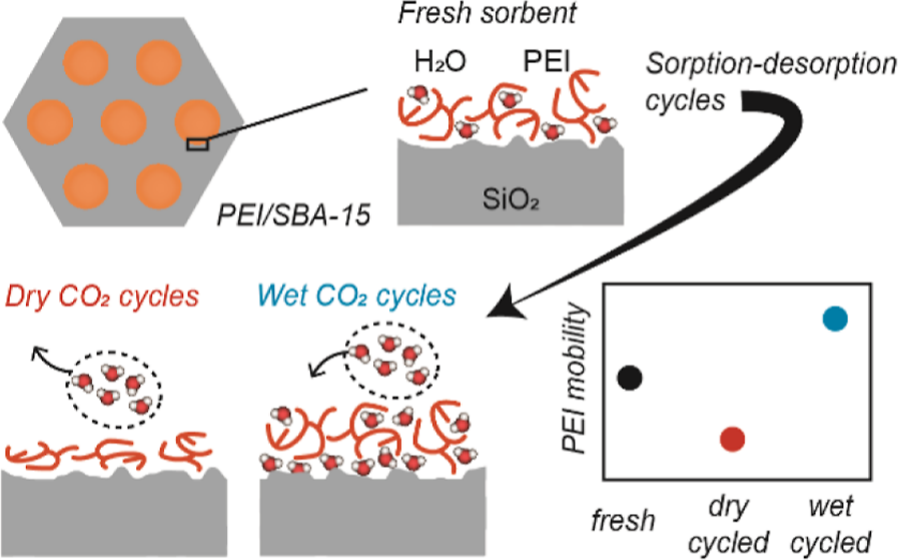

Solid-supported amines are effective CO_2_ adsorbents capable of capturing CO_2_ from flue gas streams (10–15 vol % CO_2_) and from ultradilute streams, such as ambient air (∼400 ppm CO_2_). Amine sorbents have demonstrated promising performance (e.g., high CO_2_ uptake and uptake rates) with stable characteristics under repeated, idealized thermal swing conditions, enabling multicycle application. Literature studies suggest that solid-supported amines such as PEI/SBA-15 generally exhibit slowly reducing CO_2_ uptake rates or capacities over repeated thermal swing capture-regeneration cycles under simulated DAC conditions. While there are experimental reports describing changes in supported amine mass, degradation of amine sites, and changes in support structures over cycling, there is limited knowledge about the structure and mobility of the amine domains in the support pores over extended use. Furthermore, little is known about the effects of H_2_O on cyclic applications of PEI/SBA-15 despite the inevitable presence of H_2_O in ambient air. Here, we present a series of neutron scattering studies exploring the distribution and mobility of PEI in mesoporous silica SBA-15 as a function of thermal cycling and cyclic conditions. Small-angle neutron scattering (SANS) and quasielastic neutron scattering (QENS) are used to study the amine and H_2_O distributions and amine mobility, respectively. Applying repeated thermal swings under dry conditions leads to the thorough removal of water from the sorbent, causing thinner and more rigid wall-coating PEI layers that eventually lead to slower CO_2_ uptake rates. On the other hand, wet cyclic conditions led to the sorption of atmospheric water at the wall-PEI interfaces. When PEI remains hydrated, the amine distribution (i.e., wall-coating PEI layer thickness) is retained over cycling, while lubrication effects of water yield improved PEI mobility, in turn leading to faster CO_2_ uptake rates.

## Introduction

Amine-based CO_2_ sorbents are a well-studied class of sorbents enabling CO_2_ capture from ultradilute sources, such as ambient air (∼400 ppm CO_2_). However, liquid amines are often prone to evaporation and oxidative degradation.^1,2^ To overcome such challenges, amines have been incorporated into porous solid supports, yielding a hybrid family of materials referred to as solid-supported amines.^3−18^ Among diverse types of solid-supported amines, low-molecular-weight aminopolymers physically imbibed into mesoporous metal oxides (e.g., silica and alumina) have been extensively used due to their facile and reproducible syntheses and promising CO_2_ uptake performance.^7−15^ One prototype solid-supported amine is low-molecular-weight (∼800 DA), branched poly(ethylenimine) (PEI) physically impregnated into mesoporous SBA-15 silica (PEI/SBA-15).^7−9,11^ This system has demonstrated promising CO_2_ uptake with stable performance characteristics under laboratory conditions using varied feed conditions and thermal regeneration processes, enabling multicycle applications.^19^ The use of infrared (IR) spectroscopy, NMR spectroscopy, and computational modeling have allowed for improved molecular-level understanding of the operation of these materials, such as reaction chemistries in the presence of CO_2_ and H_2_O,^20−24^ responses of the amines toward oxidative stresses,^9,25−28^ incorporating small-molecular additives^29−31^ or using mixed amine systems,^32,33^ and the effects of the pore topologies^34−37^ and surface chemistries of the solid supports,^38−40^ guiding researchers toward optimal materials design principles for this application. In addition to the chemical behavior of amines, the physical attributes of amines, such as the distribution and mobility of the amines on/off the solid supports, play crucial roles in the CO_2_ capture properties. Our recent studies showed that PEI distribution and mobility play crucial roles in determining CO_2_ capture kinetics and capacities. For instance, our small-angle neutron scattering (SANS) and solid-state NMR (ssNMR) studies suggested that there are wall-coating amines with strong affinity between amines and silanols on pore walls, restricting reorientation of amines and thereby limiting CO_2_ uptake.^41−43^ Our quasielastic neutron scattering (QENS), ssNMR, and molecular dynamics (MD) simulation studies elucidated that PEI motions are responsible for CO_2_ diffusion along amine-packed pores, which in turn influences CO_2_ uptake rates.^39,42,44^

Literature studies on PEI/SBA-15 and other solid-supported amines have reported that sorbents generally exhibit deteriorating CO_2_ loadings when repeating CO_2_ capture-thermal regeneration processes.^12,13,45−48^ Understanding the aforementioned physical attributes of the amines (e.g., amine distribution and mobility) can lead to sorbent design and process principles for more effective multicycle applications. We hypothesize that thermal regeneration or thermal swings associated with cyclic temperature-swing adsorption (TSA) applications may cause restructuring of the amines in the pores, potentially altering amine distribution and mobility. Additionally, there has been no clear experimental evidence indicating how atmospheric water impacts the structure and motions of PEI in SBA-15 pores. It is critical to understand the impact of water in repeated capture-regeneration cycles, as water may disrupt the amine distribution by hydrogen-bonding to amines and those water molecules may persist in the adsorbents over many cycles, as suggested by a recent MD simulation study.^49^

Here, with PEI/SBA-15 as a model sorbent, we investigate the effects of repeated thermal-swing sorption-regeneration cycles and the effect of humidity under such TSA conditions from the perspective of the PEI distribution and mobility within the silica support pores. Through a combination of neutron scattering studies (SANS and QENS), we show that thermal cycles together with varied humidities lead to unique PEI distributions and mobilities. Applying dry gas streams during thermal cycles resulted in the removal of water from PEI, causing rigid, thinner PEI films to form around the silica walls with reduced PEI mobility. Such effects led to slower CO_2_ sorption kinetics. On the other hand, humid cycles resulted in either retained (under moderate humidity) or slightly increased water contents (under higher humidity) in the PEI/SBA-15 sorbents, with water eventually reaching the wall-PEI interfaces not being effectively removed during the thermal regeneration steps, consistent with prior findings via MD simulation.^49^ The hydrated PEI around the walls led to a higher overall PEI mobility, resulting in faster CO_2_ uptake.

## Experimental Section

### Mesoporous SBA-15 Synthesis

SBA-15 was synthesized following the procedures described in our previous articles.^39,41^ Approximately 2.48 g of the block copolymer template (Pluronic P-123; EO_20_PO_70_EO_20_, Sigma-Aldrich) was dissolved in 82 mL of deionized water in a round-bottomed pressure flask, during which 11 mL of 12.1 M HCl (Sigma-Aldrich) was added. The solution was stirred vigorously for 2 h to achieve a homogeneous dispersion of the block copolymer template. To form porous solids, 5.8 mL of precursor (tetraethyl orthosilicate; TEOS; Acros Chemicals) was added to the solution dropwise. The reaction flask was then put to a heating bath, and the temperature was ramped to 40 °C in ∼ 1 h and then held at 40 °C for 24 h while maintaining a gentle mixing under ∼ 800 rpm during which a white precipitate was formed. The precipitate was thermally aged at ∼ 135 °C for 24 h without stirring. After the hydrothermal synthesis and aging, the reaction flask was cooled by being submerged in cold tap water. The resultant precipitate was rinsed and filtered with a copious amount of deionized water and then stored in a drying oven (∼110 °C) overnight. Lastly, the resultant powder was gently ground and calcined following the temperature program—temperature ramp to 200 °C in 1.2 °C/min rate, temperature maintained at 200 °C for 1 h, ramp-up to 550 °C with 1.2 °C/min, maintained at 550 °C for 6 h, and then cooled to ∼ 50 °C.

### Synthesis of Deuterated Poly(ethylenimine)

**[Hazard note: ethylenimine (aziridine) is highly volatile and very toxic and is a strong irritant to the respiratory system and skin. Aziridine is an alkylating agent that may induce mutation of DNA and is classified as a possible carcinogen. Use with extra caution; handle the chemical in a fume hood with proper personal protective equipment; and keep any isolated aziridine sufficiently chilled. Wash any items potentially contaminated with aziridine using mild acids such as acetic acid.]** Synthesis of poly(ethylenimine)-*d*_5_ (dPEI) followed the protocols described in our previous article,^41^ and details of the synthetic protocols can be found in the Supporting Information. Briefly describing the synthetic procedure, the deuterated precursor (ethylenimine-*d*_4_) was synthesized first via the bromination of ethanol-amine-*d*_4_ (CDN isotopes) to bromoethylamine-*d*_4_ by heating in aqueous HBr. Second, base-activated ring closure was applied to bromoethylamine-*d*_4_ to form ethylenimine-*d*_4_ (aziridine-*d*_4_), after which vacuum distillation was carried out to concentrate the ethylenimine-*d*_4_. Then, dPEI was synthesized by the acid-catalyzed ring-opening polymerization of ethylenimine-*d*_4_ (aziridine-*d*_4_) with 2HCl-coupled ethylenediamine-*d*_4_ as an initiator as well as a capping agent.

### Composite Synthesis and Characterization

For all PEI/SBA-15 samples, the polymer was incorporated in the pores of SBA-15 by physical impregnation. For dPEI/SBA-15 synthesis (for SANS samples), impregnation procedures used MeOD (Sigma-Aldrich) as the solvent to avoid exchange of the labile deuterium (e.g., ND_*x*_) with protons. For regular PEI/SBA-15 (for QENS and other measurements), MeOH (Sigma-Aldrich) was used. The polymer solution was prepared by dissolving the polymer in 10 mL of methanol (MeOD or MeOH) and stirring for 2 h. Evacuated SBA-15 silica was dispersed in methanol (100 mg silica:10 mL methanol) by sonication for ∼ 10 min, followed by stirring for 2 h. The polymer solution and the dispersed silica were combined and then mixed for 12 h to allow the polymer to diffuse into pores. The solvent was removed using a rotary evaporator, followed by evacuation under ∼ 10 mTorr in a Schlenk flask at room temperature for 72 h to remove residual CO_2_, moisture, and volatiles. Samples were stored in a N_2_ atmosphere before further analysis to ensure the health of the composites (e.g., avoiding oxidation of PEI) and to minimize H_2_O or CO_2_ uptake.

Textural properties and porosity of the composites and the evacuated SBA-15, N_2_ physisorption was conducted using a Micromeritics Tristar 3020 at 77 K. Samples were degassed at 80 °C under vacuum (∼30 mbar) for 12 h prior to measurement. Pore volume and pore size distributions were estimated by using the NLDFT equilibrium model (adsorption isotherm) using the functionality equipped in Quantachrome VersaWin software. Surface areas were determined by the BET method. Lastly, the weight fractions of PEI in the composite materials were estimated via combustion TGA measurements. A TA instruments Q500 TGA was used with N_2_ flow, ramping temperature from 30 to 120 °C in 10 °C/min ramp, holding at 120 °C to aid the removal of adsorbed water from the atmosphere, and again ramping to 700 °C in 10 °C/min ramp rate. Mass loss from 120 to 700 °C was taken as the mass fraction of impregnated PEI.

### Treatment of Composite Sorbents under Repeated CO_2_ Sorption-Regeneration Processes

The powder samples were loaded on a 250 μL ceramic TGA pan (TA Instruments) and treated with a thermal swing under flowing gas streams. For dry experiments, dry N_2_ was used for sample activation and sorbent regeneration (at 100 °C), and 400 ppm CO_2_ (balance N_2_) was applied for CO_2_ capture steps (at 30 °C). For wet cyclic experiments, the gas input was passed through a dew point generator (LICOR). The humidity was cross-checked with an IR gas analyzer (LICOR). After cyclic applications, samples were placed in a vial and backfilled with dry N_2_ prior to further evacuation processes.

### Cryo-Evacuation before Neutron Scattering Experiments

Samples were evacuated under cold temperatures to remove adsorbed H_2_O or CO_2_ while minimizing the restructuring of PEI in the pores. Samples were rapidly frozen by submerging sample vials in liquid nitrogen, after which vacuum (∼10 mTorr) was applied to dry samples. After drying, samples were opened in a He-filled glovebox before sample packing into SANS or QENS sample cells.

### SANS Sample Preparation and Data Analysis

Gas-tight aluminum holders with 1 mm path length and 1 in. diameter quartz windows were used to pack the samples (with ∼ 50 mg silica mass basis). All assembly processes were performed under a He atmosphere in a glovebox. SANS was performed on the EQ-SANS at the Spallation Neutron Source (SNS) at Oak Ridge National Lab.^50^ Two instrument configurations were used to access a wide *Q*-range and establish an incoherent background intensity for subtraction. Low *Q* data were acquired with a detector distance of 4 m and a neutron wavelength of 4 Å. High *Q* data were acquired with a 1.3 m detector distance and 1 Å neutron wavelengths. These configurations had a *Q*-uncertainty of less than 5% for all *Q* > 0.05 Å^–1^ and less than 10% for 0.025 Å^–1^ < *Q* ≤ 0.05 Å^–1^.^51^ Two pieces of the spectra (low *Q* and high *Q*) were stitched with an overlap region from 0.25 and 0.27 Å^–1^ and calibrated to absolute intensity by using a silica standard (Porasil). No adjustment for the SBA-15 packing fraction was made.

### QENS Sample Preparation and Data Analysis

The powder sample was deposited on pure aluminum foil, forming a thin film with an approximate thickness of ∼ 200 μm, after which the foil was folded to make an annular pouch. The pouch was then inserted into an aluminum QENS sample can, and an indium wire was used to seal the sample. All assembly processes were performed under a He atmosphere in a glovebox. QENS measurements were performed on the backscattering silicon spectrometer (BASIS)^52^ at the SNS at Oak Ridge National Laboratory (ORNL; Oak Ridge, TN, USA). BASIS was used in one of its standard configurations, using a polychromatic incident beam with a time-of-flight window defined by spanning the choppers set at 60 Hz frequency, providing the incoming neutron with bandwidth centered at 6.4 Å. At this setting, it provides a fine energy resolution of 3.5 μeV (at full width at half-maximum) while using Si(111) analyzer crystals covering a *Q* range of 0.20–2.0 Å^–1^ and an energy range of ±120 μeV. about the final energy.

## Results and Discussion

### Basic Physical Properties of Sorbents after CO_2_ Cycles

**Figure 1:**
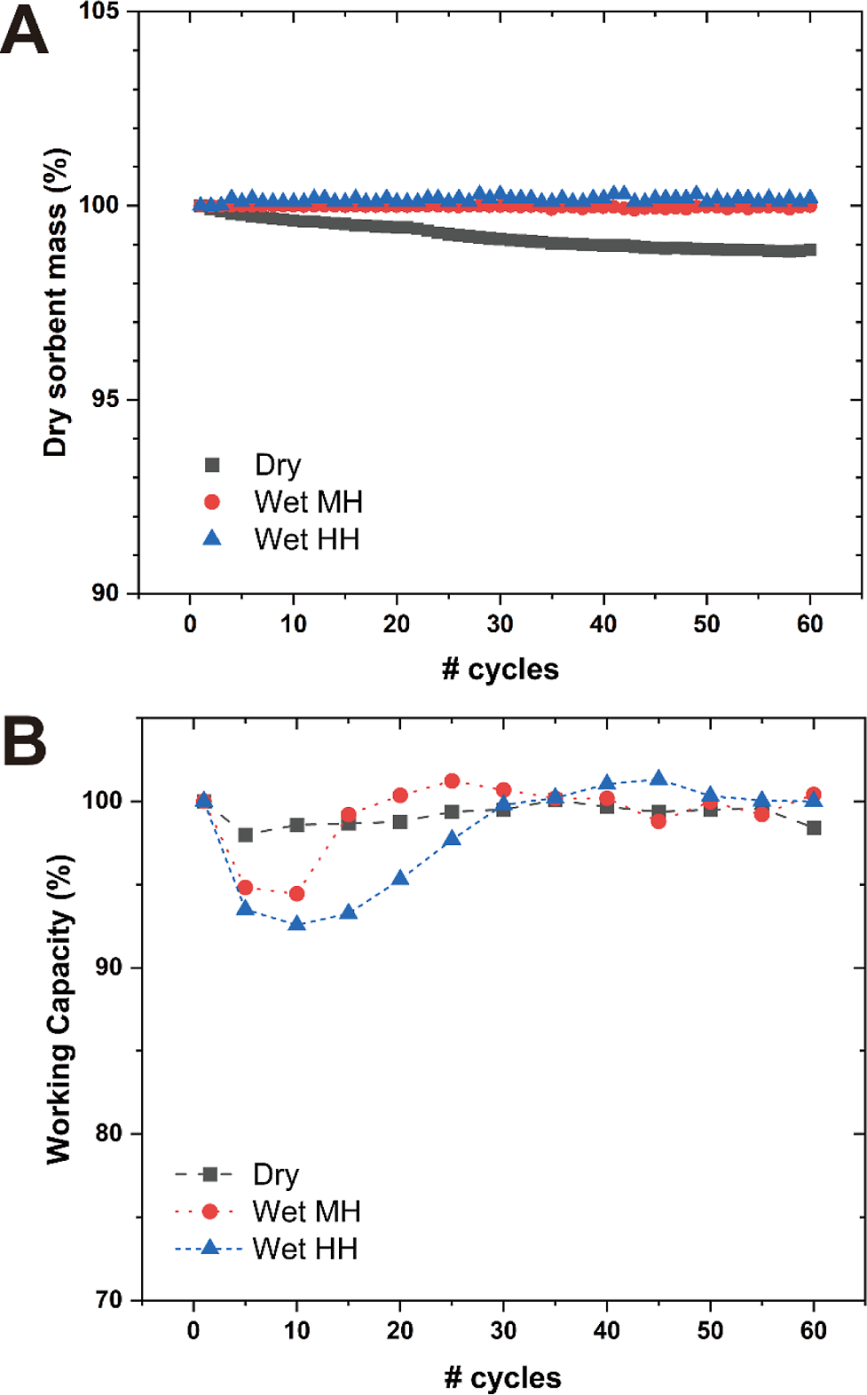
Trend of sorbent mass and working capacities through cycles. (A) Sorbent mass under dry, medium humidity, and high humidity conditions based on the sorbent mass right after regeneration. (B) CO_2_ uptake under cyclic conditions. For wet cycles, relative CO_2_ uptake values were taken based on the detected gas composition at the gas analyzer.

First, the mass of the sample was characterized after treatment under varying cyclic conditions (e.g., input gas stream and number of cycles). Figure 1A shows the sample mass right after the regeneration processes. We observed a gradual mass decrease after dry cycles, reaching an asymptote of ∼ 98.8% of the original mass (in 60 cycles). Given that the mass fraction of PEI remained intact (checked via combustion TGA, Figure S1), this small mass loss is attributed to the removal of remnant H_2_O from the adsorbent. This H_2_O may be stabilized at PEI-wall interfaces by hydrogen bonding to surface silanol groups as well as PEI. However, as samples were subjected to repeated thermal swings, with a larger extent of PEI motions at high temperatures and with purging of the sorbent with a dry gas stream, such confined H_2_O was removed under dry cyclic conditions. On the other hand, for humid cycles, as observed in Figure 1A, samples showed consistent sorbent mass [in the case of ∼ 35% relative humidity (RH) at 30 °C, ∼ 10.6 g/m^3^ absolute humidity] or a slight increase under higher humidity (∼60% RH, ∼ 18.2 g/m^3^ absolute humidity).

Again, given the consistent PEI mass observed via combustion TGA (Figure S1), the unchanged sample mass suggests retention of water from sorbent synthesis in the material at low RH and the slightly increased sample mass at higher RH indicates a slight accumulation of H_2_O. Upon characterization of the CO_2_ working capacities, Figure 1B shows consistent uptake for the dry cycles (98–100% compared to the first cycle), while wet cycles show a noticeable fluctuation but eventually recover to an uptake comparable to that of the first cycle. Interestingly, different humidities yield different extents of fluctuation, with a relatively shallow fluctuation under medium humidity and more pronounced variation under higher humidity. We suspect that the decreased working capacity at the early stage may be attributed to H_2_O occupying pore volume in open pore spaces (e.g., around pore mouths or pores unoccupied with PEI). This trend was not observed for the sample treated under medium humidity, which did not show noticeable mass increase due to the addition of water. However, there could be redistribution of H_2_O molecules in the materials over cycling, for example, building clusters or occupying a different part of the pore space, even in cases where there was little cycle to cycle mass change. A similar trend was captured by previous MD simulation articles.^49,53^ However, as the system reached an apparent steady state over cycling, we hypothesize that H_2_O molecules find the most stable dispersion after a sufficiently large number of cycles.

### Effects of Cyclic Applications on PEI Distribution and Mobility

As discussed previously, we observed that cyclic applications affected the sample mass and CO_2_ uptake. Understanding the underlying physical properties of PEI before and after cycling can help elucidate the basis of such altered behavior. Above, we discussed the potential removal or inclusion of H_2_O, both of which may affect PEI distribution and mobility. Less hydrated PEI may be more brittle than hydrated PEI, and different extents of hydration of the pore surfaces may give distinguishable PEI structures at the interfaces. To effectively characterize the PEI in the silica pores, we deployed neutron scattering, as neutrons interact more strongly with organic PEI than the inorganic silica support, giving useful insights into our PEI/SBA-15 systems. In particular, we used SANS to assess the PEI distribution. To characterize PEI mobility, QENS was used. To aid in interpretation, we prepared samples treated under varied cyclic conditions, and the results are listed in Table 1. We note that samples for SANS contained fully deuterated PEI (dPEI) to enhance neutron contrast against the silica support while minimizing incoherent scattering from ^1^H. For QENS samples, hydrogen-rich PEI (following natural abundancy; ∼ 99.99% ^1^H) was used to maximize the incoherent scattering that encodes the dynamic properties of the PEI molecules.

**Table 1:** List of Samples Investigated via Neutron Scattering^c^

SANS samples: ∼ 40 wt % dPEI/SBA-15^a^				
sample name	pristine	Dry (10/30/60)	Wet MH (10/30/60)	Wet HH (10/30/60)
description	as-synthesized	cycled under dry gas stream	cycled under medium humidity	applied high humidity
		(dry N_2_ or CO_2_/N_2_)	(10.6 g/m^3^ H_2_O)	(18.2 g/m^3^ H_2_O)

QENS samples: ∼ 40 wt % hPEI/SBA-15^b^				
sample name	pristine	Dry 60	Wet MH 60	Wet HH 60

a dPEI: deuterated PEI.

b hPEI: PEI with ∼ 99.99% ^1^H.

c Sample names are shortened as follows—Dry10 (or 30 or 60) representing dry-cycled samples for 10 (or 30 or 60) cycles, Wet MH10-60 for wet cycles with medium humidity, and Wet HH10-60 for high humidity.

#### Tracking PEI Distribution via SANS

As mentioned earlier, dPEI was used to obtain a clear neutron contrast between PEI and SBA-15. This can also minimize the extent of incoherent scattering resulting from ^1^H (which flattens the SANS spectrum at high *Q*) and its use is therefore imperative to ensure the observation of diffraction peaks. The estimated neutron scattering length density (SLD) of dPEI was ∼ 8.2 × 10^–6^ Å^–2^ (calculated based on atomic properties),^54^ whereas the amorphous SiO_2_ phase of SBA-15 has a neutron SLD of ∼ 3.5 × 10^–6^ Å^–2^,^41^ suggesting a clear contrast between PEI and silica walls. The presence of H_2_O in samples may complicate comprehending SANS spectra, but we note that the amount of H_2_O was marginal, considering the mostly constant polymer mass fraction and mostly consistent sample mass. Furthermore, H_2_O has negative neutron SLD (∼−0.56 × 10^–6^ Å^–2^), giving a clear contrast against both silica and dPEI.

**Figure 2:**
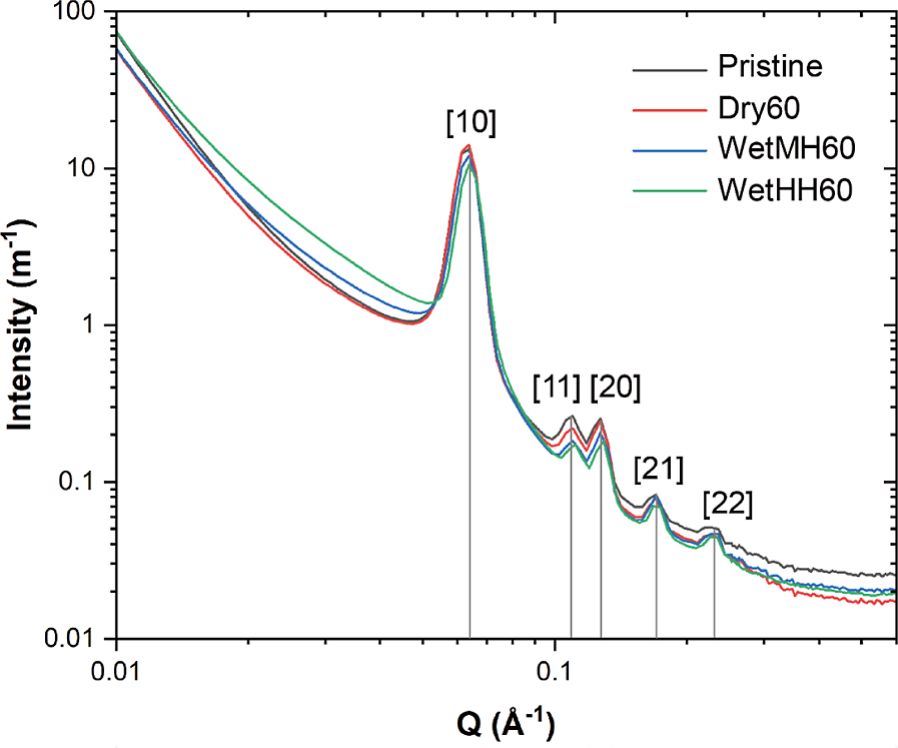
SANS spectra for samples treated under different cyclic conditions. Bragg peaks [10], [11], [20], [21], and [22] are annotated.

The representative set of SANS spectra for the as-synthesized sorbent and the sorbents that underwent 60 cycles with varied conditions (Dry 60, Wet MH60, and Wet HH60) is plotted in Figure 2. SANS spectra for all samples can be found in Figure S4. A quick trend can be observed by comparing Bragg peaks ([10], [11], [20], [21], and [22]). These Bragg peaks denote arrays of mesopore spaces surrounded by condensed silica phases, consistent with the literature.^55−57^ Upon comparing the Bragg peaks in the SANS spectra, one notes that the diffraction peaks were conserved regardless of the varied cyclic conditions, indicating that the skeletal backbone of SBA-15 remained intact. However, there were changes in peak intensities, which represent the varied distribution of nonzero neutron SLD contributors (e.g., dPEI and H_2_O). First, a trend can be observed in the intensities for peak [10]. The sample cycled under dry conditions for 60 cycles (Dry 60) had a slightly more intense [10] peak than the other samples. Comparing peaks [11] and [20], the ratios of the peak intensities changed with different cyclic conditions. The pristine, as-synthesized sample showed comparable peak intensities, *I*([11]) ∼ *I*([20]). Treating under dry conditions led to a slight decrease in peak [11] with maintained intensity in peak [20], causing *I*([20])/*I*([11]) to be larger than that of the pristine sample. Wet cyclic treatments yielded more severe decay in the [11] peak intensity compared to that of the dry case, resulting in much larger *I*([20])/*I*([11]). Altered relative peak intensities also denote changes in PEI and H_2_O distribution in the pores, which will be understood by fitting SANS spectra against theoretical models.

**Figure 3:**
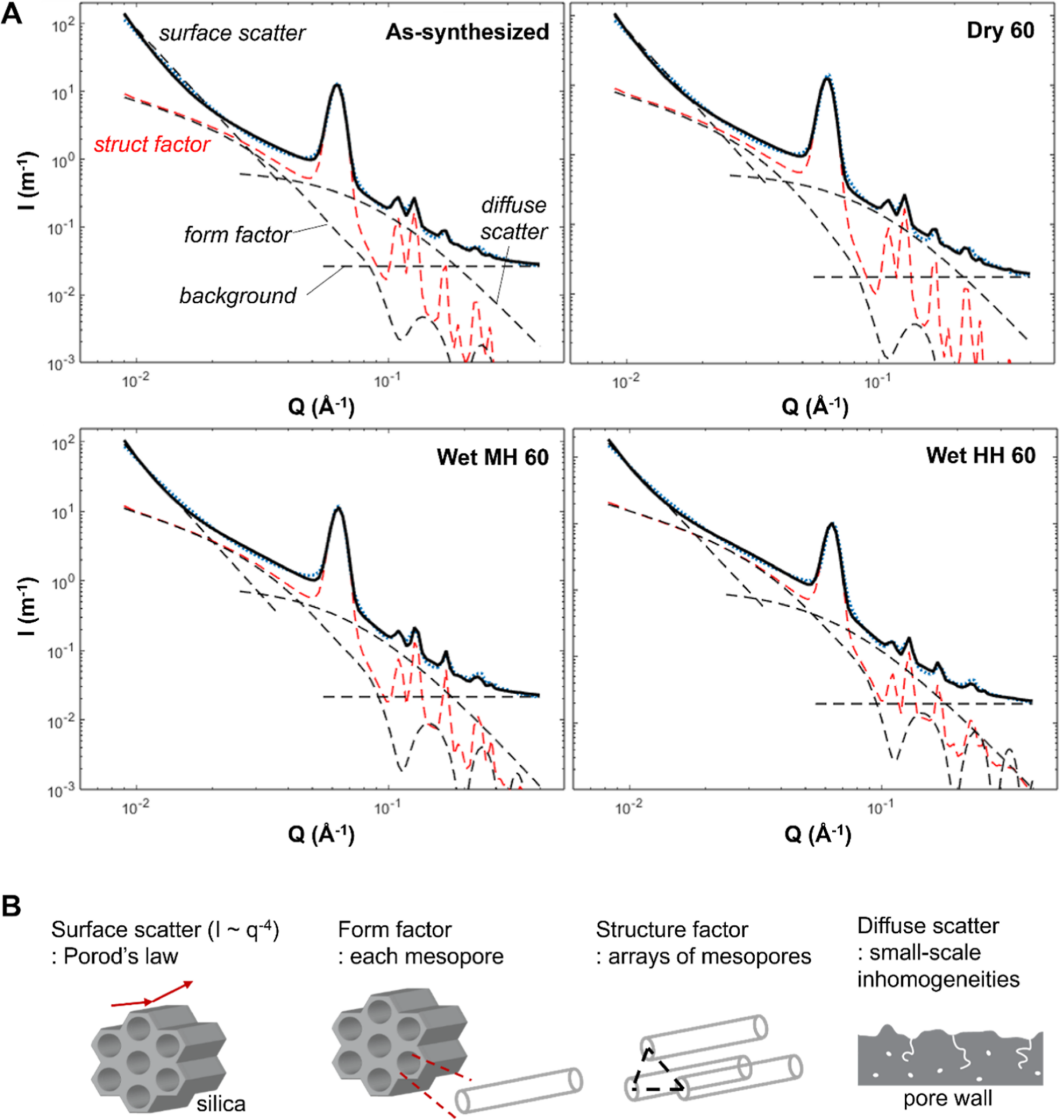
(A) Representative SANS spectra fitting results for the as-synthesized sample and samples after 60 cycles under varied streams (Dry, Wet MH, and Wet HH). (B) Schematic illustrations of structural contributors. (B) Was reproduced with permission from ref (58). Copyright 2023 American Chemical Society.

Further analyzing the SANS spectra yields more rich information. Figure 3A shows examples of SANS spectra and fitted curves for Dry 60, Wet MH60, and Wet HH60 samples. There are multiple contributors comprising the scattering law, and they are marked with dotted lines. Those contributors have their own meaning, and schematic illustrations are presented in Figure 3B. Each contributor is drawn in different *q* ranges, representing structural contributions at different characteristic length scales (), where *L* is the length in the real space. The first rapid decay observed at low *q* can be well represented by Porod’s law (*I* ∼ *c*_p_*q*^–4^), where *c*_p_ is Porod’s law constant. This is linked to the external surfaces of silica particles characterized by large length scales (Figure 3B, surface scatter). Samples showed comparable slopes and intercept, suggesting that SBA-15 morphologies remained intact. Second, the black dotted line spanning the whole *Q* range is the form factor, related to the dimensions (i.e., pore size, pore lengths, and fill fraction) and neutron SLD distributions (i.e., presence of dPEI or H_2_O) in a single mesopore domain (Figure 3B, form factor). Third, the red dotted curve is the structure factor representing the hexagonal arrays of mesopores in a silica particle. The peaks show consistent peak positions (i.e., hexagonal pore arrays remained intact; Figure 3B, structure factor) but with different intensities (i.e., potentially varied arrangements of dPEI in pores). Fourth, there is a dotted line with a smooth decay starting at medium *Q*, and that represents diffuse scattering addressing small-scale structural inhomogeneities, such as intrawall pores, occluded pores, and corrugated surfaces (Figure 3B, diffuse scatter). Lastly, there is a flat dotted line (background). With the aforementioned functions, and with several parameters predetermined (e.g., pore size, pore lengths, and pore fill fraction), unknown structural parameters could be estimated, following the methodology from our previous work^41^ (details in the Supporting Information).

As discussed above, the SANS spectrum encodes rich structural information, and curve fitting yields quantitative structural parameters such as the volume occupied by PEI, the thickness of the wall-bound PEI film around the pore walls, the neutron SLD values of the corona layer, and so on. The mathematical expression of the overall SANS spectrum is denoted in eq 1.
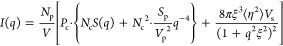
1Here, *N*_p_ is the number of particles in a sample cell, *V* is the volume of the sample cell, *P*_c_ is the form factor of the mesopore (with or without PEI and H_2_O), *S*(*q*) is the structure factor, *N*_c_ is the number of unit cells, *S*_p_ is the external surface area of a silica particle, ξ is the correlation length along the pore walls (which deals with permeation of PEI chains or H_2_O along smaller, intrawall pores), ⟨η^2^⟩ is the SLD fluctuation (⟨η^2^⟩ = ⟨ρ^2^⟩ – ⟨ρ⟩^2^), and *V*_s_ is the solid skeletal volume (i.e., volume of the nonporous region of a silica particle). Details, such as the derivation, can be found in the Supporting Information.

Fitting the observed SANS data against theoretical models yielded structural parameters useful for understanding the PEI distribution and the presence of H_2_O around the pore walls, as listed in Table 2. Fit parameters for all other samples can be found in Table S1. The first row represents the distribution of dPEI on the mesoscale. This could be determined by the form factor that best describes the observed SANS spectrum. Three types of models were used—core–shell (polymer layers growing from wall-coating polymer domains), plug (polymer aggregates growing from pore centers with marginal wall-coating domains, with minimal wall-coating polymer domains), and sequential model (core–shell and then plug, with a varied extent of polymers in pore-coating layers). More details, such as the mathematical expression of the models and potential limitations, can be found in the Supporting Information. As shown in Table 2, the pore-coating domain occupied ∼ 20 vol % in the as-synthesized sample. Applying dry streams for 60 cycles decreased the pore-coating domain to about half this volume fraction (∼10 vol % pore-coating). On the other hand, wet cycles led to retained pore-coating volume fraction, comparable to that of the fresh sample. More rich information could be derived from the structural parameters listed in Table 2.

**Table 2:** Structural Parameters Extracted by SANS Curve Fit

	evacuated SBA-15	PEI/SBA-15 as-synthesized	Dry 60	Wet MH 60	Wet HH 60
model	–	20 vol % shell	10 vol % shell	20 vol % shell	20 vol % shell
*R*_p_ (Å)	38	34	36	34	35
*t*_c_ (Å)	10	12	6	14	12
corona SLD	0.35	0.8	0.8	0.5	0.15

Next, we can compare length scales that correspond to polymer plugs and pore-coating layers. The radius of the polymer plug coexisting with the available void space (*R*_p_) and the thickness of the polymer-bound pore walls (*t*_c_) can be compared. Upon comparing *R*_p_ and *t*_c_ for dPEI/SBA-15 samples, we observe that *R*_p_ showed consistent values, while Dry 60 had *t*_c_ significantly lower than those of the other samples. This suggests there are potentially thinner dPEI layers around the walls in Dry 60 sample compared to other dPEI/SBA-15 samples. We then focus on the wall-polymer interfaces. We define the domain that corresponds to the polymer-wall interfaces (within the length scale of *t*_c_) as a corona layer, where there are SiO_2_ matrices, impregnated dPEI, and potentially H_2_O populated around the pore walls. In the corona layer, there are complex structural features, including intrawall micropores and occluded pores with corrugated surfaces. Adding polymers and H_2_O makes the corona layer more complicated. Varying the neutron SLD within the corona layer (third row in Table 2; corona SLD) and fitting against the observed SANS spectrum, plausible SLD values were extracted and compared. Comparing samples treated for 60 cycles, we noticed a significant decrease in the corona SLD for wet-cycled samples, while the dry-cycled sample has a corona SLD consistent with that of the as-synthesized sample.

**Figure 4:**
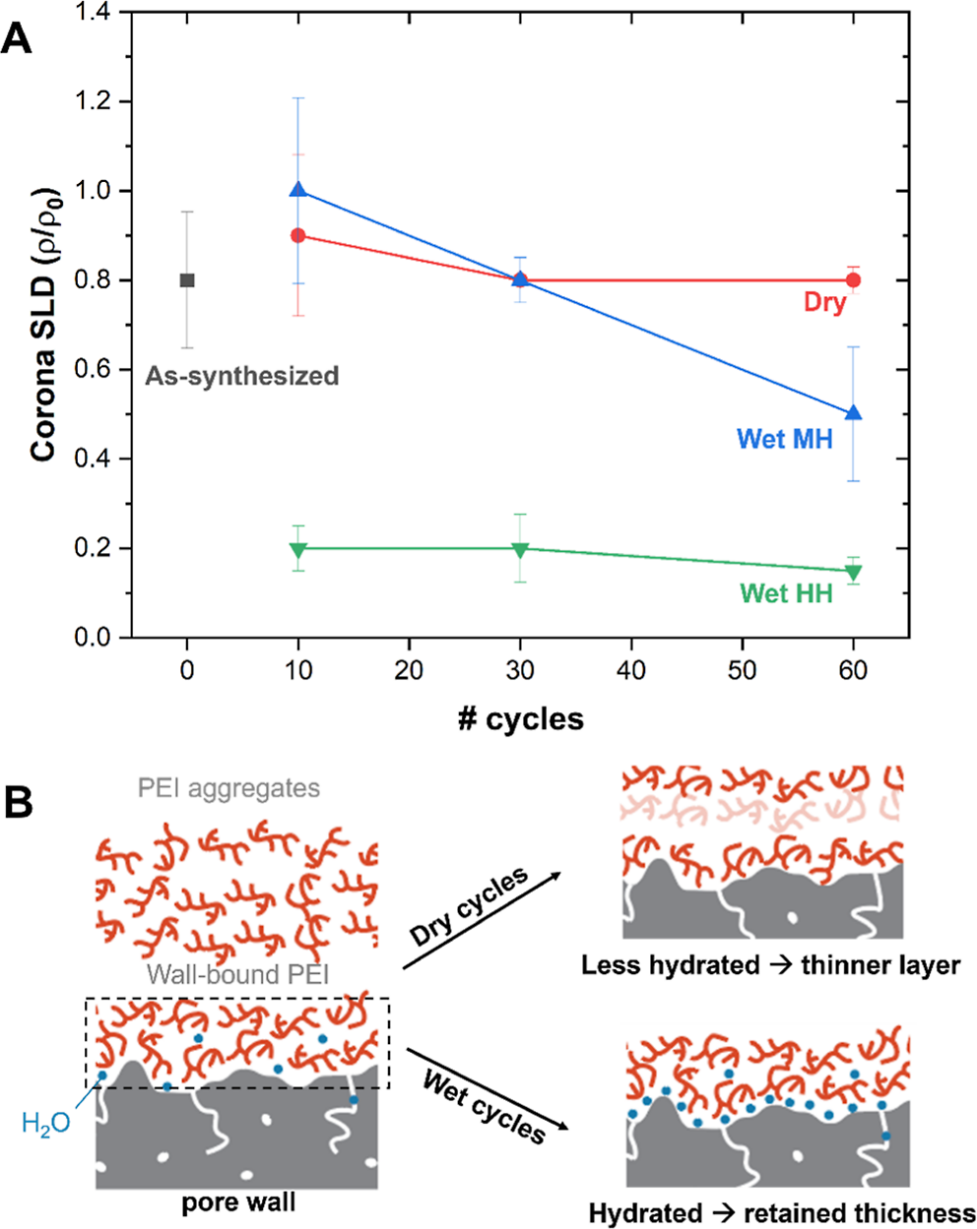
Hypothesized structures at pore wall-PEI interfaces. (A) Corona SLD as a function of cyclic conditions. Error bars represent standard deviations from three best fit results. (B) Hypothetical distribution of PEI and H_2_O around pore walls.

Figure 4A shows a trend of corona SLD as a function of cyclic conditions and the rationalization of the varied SLD values. Wet cycles caused evident lowering of the corona SLD, while dry cycles led to retained values. The lowered corona SLD was attributed to the addition of H_2_O, which has negative neutron SLD. Comparing the effects of varied humidities, a higher humidity caused more profuse H_2_O penetration along the PEI, leading to a dramatic SLD decrease even after a small number of cycles. Our findings suggesting H_2_O penetration toward the wall-PEI interfaces agree with our recent MD simulation studies on PEI/MCM-41 silica.^49^ We note that the MD simulation dealt with the equilibrium state, not cyclic conditions, and therefore, those results do not perfectly correspond to cyclic conditions. However, we consider that thermal regeneration in our cyclic studies could push any H_2_O molecules remaining in the sorbents to their most favorable state, potentially allowing them to find their equilibrium or near equilibrium states. Figure 4B shows a summary of our findings via SANS. The corona layer thicknesses and corona SLD collectively suggest the same trend. The H_2_O bound closer to walls (unchanged sample mass under medium humidity or potentially some added H_2_O under high humidity) help maintain the corona layer thickness, as H_2_O could moderate the pore surface-PEI or PEI–PEI binding. On the other hand, dry cycles caused dehydration of the sample by the removal of H_2_O that remained from sorbent synthesis. Thinner PEI layers around the walls can be explained by less hydrated walls or formation of patchy PEI clusters.

We also considered that losing H_2_O in the corona layer could enhance the corona SLD and tried higher SLD values for the SANS data fitting; however, these did not result in better fits to the experimental data. We hypothesize that less hydrated PEI may be less swollen due to less intercalated H_2_O interrupting the interamino hydrogen bonding in the branched PEI chains. This could result in a noticeable decrease in PEI packing around the walls, making potentially thinner PEI films with more significant open pore spaces.

#### PEI Mobility after Cyclic Applications

As discussed above, different cyclic conditions led to altered PEI distributions induced by the varied amounts and distributions of H_2_O in the sorbents. Such differing extents of hydration may in turn affect PEI mobility. To investigate this, QENS experiments were carried out. An energy window and *q* range were chosen to cover suitable time (*ps* ∼ *ns*) and length scales (3 ∼ 20 Å) relevant to PEI motions.^52,59^ A detailed expression describing the resolvable PEI dynamics via QENS is placed in the Supporting Information, where QENS utilizes incoherent scattering, in contrast to SANS, which gathers information from coherent scattering. The incoherent scattering can produce self-correlation functions, which encode dynamics. Hydrogen yields significantly larger incoherent scattering compared to any other atoms. For this reason, regular PEI (with ∼ 99.99% ^1^H) (or hPEI) is preferred instead of the dPEI used in SANS experiments. Therefore, hPEI/SBA-15 composites were used for QENS studies. To minimize incoherent scattering from the adsorbed water under humid conditions, D_2_O was used for humid streams.

**Figure 5:**
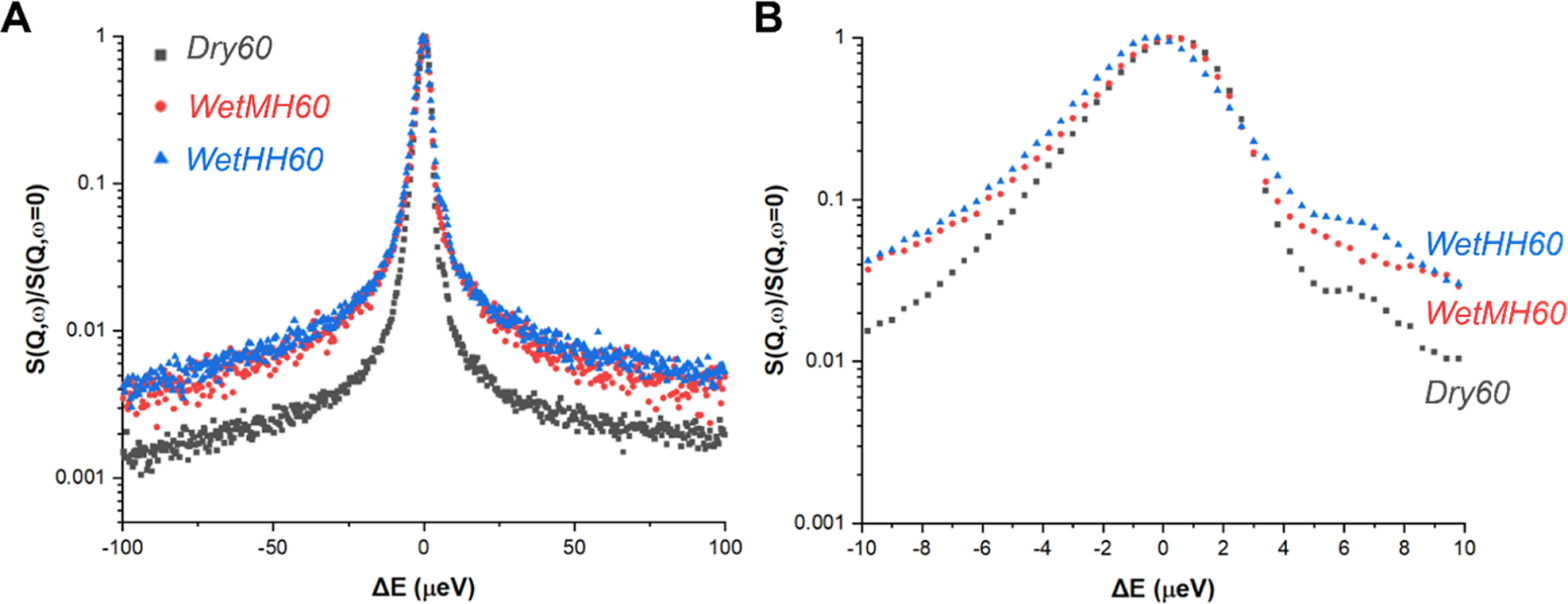
(A) QENS spectra for samples treated under different cyclic conditions. (B) Highlighting QENS broadening at the lower energy window representing slower, center-of-mass diffusion of the PEI mass. Spectra were recorded at 360 K.

Figure 5 shows QENS spectra, which elucidate the trend in PEI mobility after cyclic applications. Broader QENS spectra can be associated with more mobile systems, characterized by higher intensities at larger extents of energy transfer. Figure 5A shows QENS spectra covering the whole energy transfer window, and Figure 5B highlights the QENS broadening at the lower energy window (|Δ*E*| ≤ 10 μeV), which corresponds to slower, center-of-mass diffusion type PEI motions. The larger energy transfer range correlates to faster, localized motions such as motions of branched chains. In Figure 5A, dry cycles gave evidently narrower QENS compared to wet-cycled cases, suggesting less mobile PEI. This could be due to the extensive removal of water from the systems, lessening the lubrication effects of water intercalated to PEI domains. Comparing the wet-cycled cases, the sample exposed to higher humidity (∼18.2 g/m^3^) showed a slightly broader QENS spectrum compared to that exposed to moderate humidity (∼10.6 g/m^3^), indicating slightly higher PEI mobility after cycling under higher humidity.

**Figure 6:**
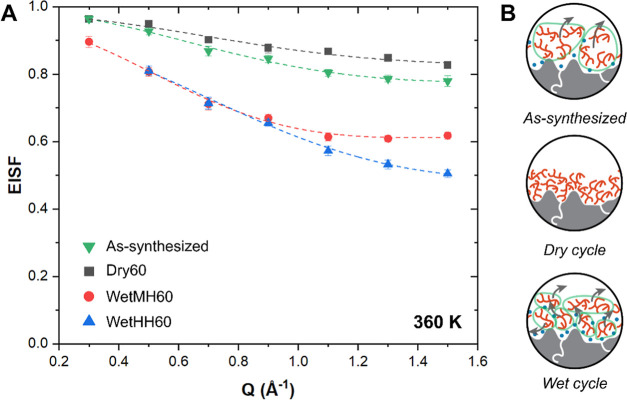
(A) EISF plots for the fresh sample and samples treated under different cyclic conditions, measured at 360 K. Dotted lines denote EISF fits to the theoretical model (eq 3). (B) Hypothesized structures and motions of PEI under different extents of intercalated H_2_O (upper: as-synthesized, middle: dry-treated, and lower: wet-treated).

To gain more detailed information, the elastic incoherent structure factor (EISF) was calculated, which encodes the geometries of the motions. The maximum of the EISF is unity, where all scattering events happened in an elastic manner (i.e., no noticeable energy transfer detected), and the EISF for truly mobile systems can approach zero. For most systems, EISF finds an asymptotic value at the high *q* limit, suggesting the extent of mobility. Figure 6 shows the EISF as a function of *q* values for four different cases (as-synthesized Dry 60, Wet MH60, and Wet HH60) at 360 and 375 K. EISF plots at other temperatures (330 and 345 K) can be found in Figure S5. PEI mobility can again be compared based on the asymptotic values at the high *q* range, suggesting a similar trend as seen in the QENS broadening. In addition, the extent of mobility at various length scales can be taken from the EISF plots. The *q* values on the *x*-axis directly relate to the length scale of the motions, following the relationship shown below (eq 2).
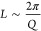
2

Comparing the dry cycled sample against the as-synthesized sample, we notice an EISF deviation at low *q* (∼0.5 Å^–1^; corresponds to *L* ∼ 13 Å) compared to the as-synthesized sample. This is relatively larger compared to the length scale of a PEI molecule of ∼ 5–8 Å, suggesting global, diffusive motions.^44^ We consider that decreased PEI mobility at a large length scale can be attributed to more rigid PEI around the pore walls in the absence of water. Furthermore, the EISF showed a larger deviation from the as-synthesized sorbent at a high *q* range (*q* > 1.1 Å^–1^; corresponds to *L* < 6 Å), suggesting less mobility associated with local PEI motions, for example, branched chain motions. Pivoting to wet-cycled samples, we captured much faster EISF decay in the low *q* region (∼0.5 Å^–1^) than that for the as-synthesized and dry-cycled samples, suggesting improved global, diffusive PEI mobility. We consider that hydration of PEI at the walls could induce more active PEI motions at the PEI-wall interfaces. Such effects could in turn enhance the overall PEI mobility in the mesopores—any wall-bound PEI can coordinate to neighboring PEI, and fast-moving wall-bound PEI can yield better PEI mobility in the overall pore spaces. Comparing the effect of humidity, the sample exposed to higher humidity showed more EISF decay, reaching a lower EISF asymptote. The EISF for high humidity showed a departure from the medium humidity case at *q* ∼ 0.9 Å^–1^ (*L* ∼ 7 Å), about the length scale of the PEI molecules. We anticipate that higher humidity brought about a significant hydration of PEI around the walls, breaking interchain amine–amine anchoring, eventually facilitating small-scale motions.

Fitting the EISF data against a theoretical model yields the PEI dynamic parameters. The scattering law can be expressed as eq 3.
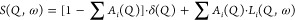
3Here, ω stands for energy transfer, δ(*Q*) is a delta function, *L*_*i*_(*Q*,ω) denotes Lorentzian functions, and *A*_*i*_(*Q*) represents spectral weights for different Lorentzian functions. From our previous papers, we found that two Lorentzian functions best represent QENS spectra in similar systems. Next, based on the same procedures used in our previous studies (derivation in the Supporting Information), the EISF can be expressed following eq 4.
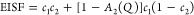
4where *c*_1_ and *c*_2_ represent the fraction of immobile scatterers to each process (*c*_*i*_ corresponds to *A*_*i*_), and the term [1 – *A*_2_(*Q*)] represents the EISF of the fast dynamic process (i.e., larger QENS broadening). To obtain the dimension of the motions, we used the spherical Bessel function of the first kind: , which describes motions happening in a spherical dimension with the radius of *R*_0_.

Table 3 shows a summary of EISF fit parameters collected at 360 K as an example (parameters for other temperatures are in Table S2). We first focus on fractions *c*_1_ and *c*_2_ for slower and faster motions, respectively. Treating the sample under dry conditions yielded a *c*_1_ value comparable to the as-synthesized sorbent, while *c*_2_ showed a noticeable increase (∼7.6%). This implies that the larger-scale diffusive mobility of PEI was largely retained but fast local motions were diminished. This agrees with the observation via SANS, where we observed less hydration of pore-coating PEI, causing a thinner PEI film on the pore walls. The dehydrated PEI film could have chains closely coordinated, causing lower local mobility. On the other hand, wet cycling reduced both *c*_1_ and *c*_2_. This is ascribed to the hydration of PEI around walls, lubricating PEI motions. Higher humidity led to a more pronounced *c*_1_ and *c*_2_ decrease, suggesting stronger lubrication effects. Next, confinement lengths (*R*_0_) characterize length scales, where PEI local motions occur. All samples showed comparable confinement lengths that correspond to the molecular size of the branched PEI, suggesting largely unchanged PEI local dynamics.

**Table 3:** EISF Fit Parameters (*T* = 360 K)^a^

*T* = 360 K	pristine	Dry 60	Wet MH 60	Wet HH 60
*c*_1_	0.99	0.98	0.95	0.91
*c*_2_	0.79	0.85	0.64	0.53
*R*_0_	2.64	2.42	3.23	2.37
*R*^2^	0.993	0.971	0.989	0.993

a *c*_1_ and *c*_2_ are fractions of immobile scatterers for slow and fast PEI motions, respectively. *R*_0_ is the confinement length and *R*^2^ values denote goodness of fits.

Quantitative dynamic parameters can be acquired by analyzing the QENS spectral widths. We addressed QENS broadening by a linear combination of two Lorentzian functions having distinct half-width at half-maximum (HWHM) values that represent the QENS broadening. A narrower Lorentzian function is correlated to slow, global PEI motions (i.e., center-of-mass diffusion), while a broader one is associated with fast, local motions (i.e., motions of branches or fast rotational motions). The Lorentzian function can be expressed as eq 5.
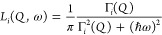
5Here, Γ_*i*_(*Q*) is the HWHM, with smaller and larger HWHM values representing slower and faster motions, respectively. The dependence of HWHMs to *Q*^2^ could be addressed by a jump-mediated diffusion model (eq 6), by which we could obtain dynamic parameters such as diffusivity (*D*), time scale (τ), and jump length [following relationship (*D* = ⟨*L*^2^⟩/6τ)].
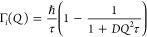
6

Figure S6 shows plots for HWHM against *Q*^2^ at two different temperatures (360 and 375 K) and corresponding curve fits against the diffusion model (eq 5). The dynamic parameters extracted from the curve fits are listed in Tables S5 and S6. Here, we focus on the time scale of motions (the rest of the dynamic parameters discussed in the Supporting Information). As eq 5 suggests, the asymptotic HWHM value at the high *Q* limit seen in Figure S6 represents the time scale of motions (as 1 + *DQ*^2^ ≫ 1). For other dynamic parameters (jump length, <*L*>, and diffusivity, *D*), we note that those two parameters are extracted from the low *Q* data [where the 1/(1 + *DQ*^2^) term in eq 5 is not negligible]. Given the large uncertainty in the low *Q* data in our systems, extracted jump lengths and diffusivities will likely not be accurate, and we consider they are only meaningful in terms of their order of magnitude. After cyclic applications, the asymptotic HWHM values and calculated timescales in Figure S6 and Table S5 and S6 suggest that dry cycles led to longer timescales than wet cycles, which again bolsters the discussion on the spectral weight analyses (EISF vs *Q*) mentioned earlier. The effect of cyclic applications was more evident in the slower PEI motions (suggested by large deviations of HWHM vs *Q*^2^ curves). This suggests that the inclusion of water may interrupt wall-PEI coordination with marginal effects on interchain motions in a PEI molecule.

### Linking CO_2_ Uptake Properties to PEI Distribution and Mobility

**Figure 7:**
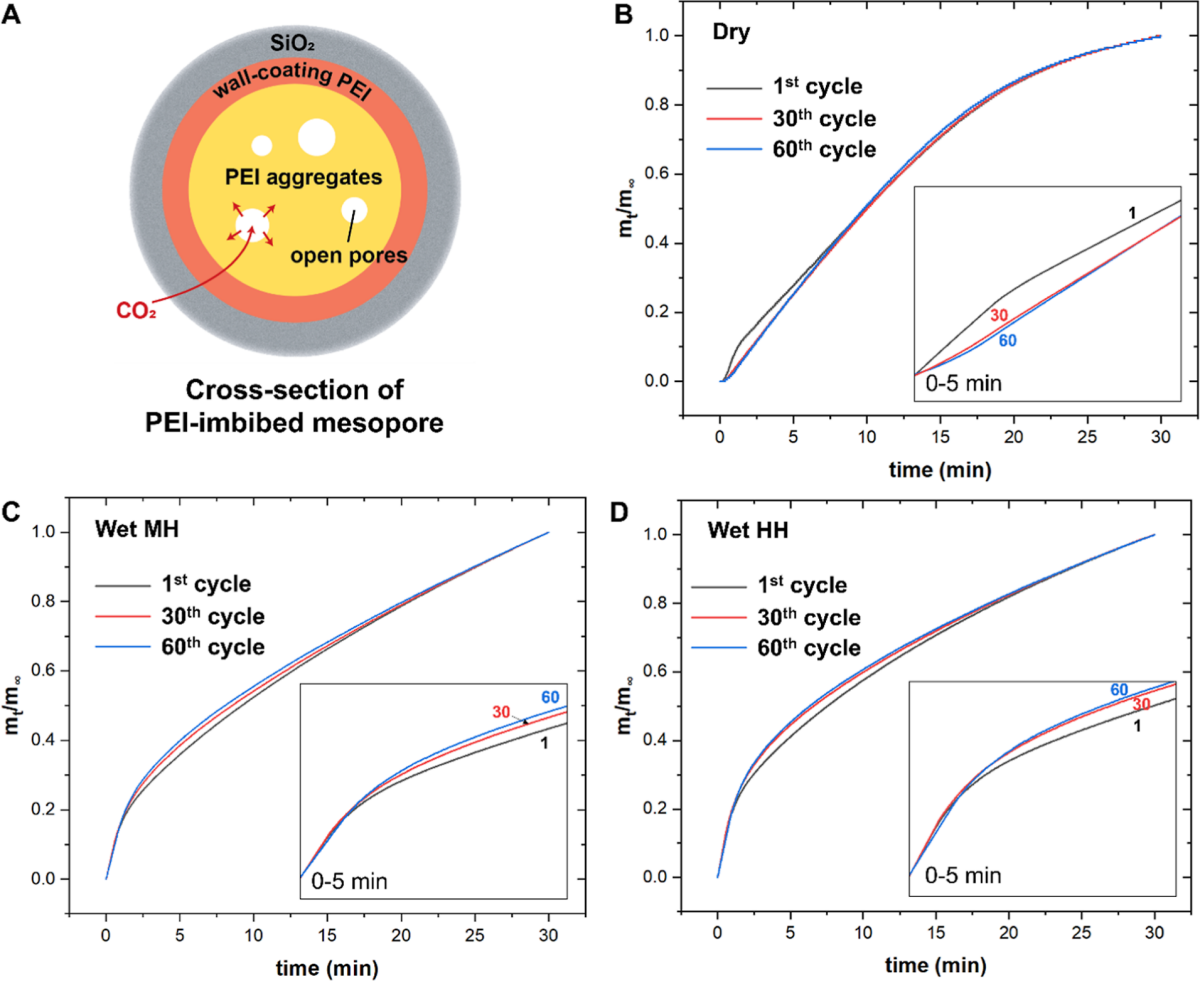
(A) Schematic of the cross-section of a mesopore in PEI/SBA-15 composites and hypothesized CO_2_ diffusion path in PEI/SBA-15 sorbents. (B–D) Fractional uptake vs time for Dry, Wet MH, and Wet HH cycles, respectively (inset graphs denote 0–5 min).

Figure 7 shows the expected CO_2_ sorption path within PEI/SBA-15 and the observed fractional uptake as a function of time, from which we can rationalize the experimental CO_2_ uptake rates. Though the samples showed comparable CO_2_ uptakes as discussed earlier (Figure 1), repeated cycling caused noticeable deviations in uptake rates. Figure 7A shows hypothetical diffusion paths of CO_2_ through the capture processes. CO_2_ enters mesopores and pore mouths, diffuses relatively rapidly through void spaces (not occupied by PEI), and first interacts with amines around void spaces. Any further CO_2_ sorption reaction requires additional amines, accompanied by CO_2_ diffusion through the PEI phase (slower process than diffusion through void). Such distinct diffusion pathways generally result in a first rapid CO_2_ sorption at the early stage, followed by a gradual uptake toward pseudoequilibrium. Figure 7B shows a comparison of uptake curves for dry-cycled samples under a varied number of cycles. The uptake rates became slower with more cycling. This can be explained by the PEI distribution and mobility elucidated by neutron scattering, as discussed in earlier sections. The dehydration of PEI created less mobile PEI, domains lowering the CO_2_ diffusivity through the PEI phase. Additionally, the uptake curve at the first cycle showed a steep uptake at the very early stage (0–2 min), which was less evident at later cycles. This may be attributed to the redistribution of PEI during thermal swings, potentially lowering the extent of open pores (i.e., voids) in the PEI domains. On the other hand, wet cyclic conditions led to faster CO_2_ uptake with increasing number of cycles, as depicted in Figure 7C,D. All samples showed an evident mass jump at the early stage (0–2 min), like the as-synthesized sorbent, suggesting that having hydrated PEI potentially retained open pores, or water lubricated PEI motions such that CO_2_ diffusivity could be retained.

## Conclusions

Solid-supported amines show altered CO_2_ capture properties under repeated capture-regeneration cycles. To gain physical insights into the underlying causes of such changes, we studied the PEI distribution and mobility via SANS and QENS with PEI/SBA-15 as a model system. It was revealed that applying dry gas streams throughout repeated capture and thermal regeneration cycles led to the dehydration of PEI domains in the mesopores of SBA-15. PEI layers around the walls became thinner (indicated by SANS), as the removal of water around pore walls caused more open silanol sites on the pore walls that could interact with PEI amine groups. The dehydration of PEI domains as well as a tight PEI-wall coordination created “stiff” PEI domains in the pores, characterized by lower PEI mobility (characterized by QENS), leading to a higher CO_2_ diffusion resistance, observed by slower CO_2_ uptakes with more cycles. Contrary to dry streams, wet cycles resulted in either a balanced (under moderate humidity) or slightly increased (under high humidity) water content in PEI/SBA-15 systems. Maintaining the hydration of PEI led to a retained PEI distribution, represented by a consistent thickness of PEI layers around the support walls. However, the data suggest that water lubricates the PEI–PEI interfaces and PEI-wall interfaces, thereby giving higher PEI mobility.

This study suggests potential areas of further study. First, gaining a clearer understanding of the distribution and binding strength of H_2_O at the support walls can give us deeper insights into the sorbents’ behavior under humid gas streams. Second, the impacts of water left over after cyclic applications can be assessed in terms of the energy economics for sorbent regeneration, as an increasing amount of water in the sorbents causes larger heat capacities for the sorbents. For our PEI/SBA-15 system, we observed only a marginal amount of water accumulation during wet cycles. However, applying a larger number of cycles or higher humidity may leave behind a larger amount of water. Other supported amine systems having porosities or cavities that can accommodate more water than other amines may have more pronounced water accumulation over wet cycles. Third, the affinity of pore walls toward H_2_O as well as PEI can determine the sorbents’ response to cyclic applications. Surface properties for SBA-15 can be modified, for instance, by having heteroatoms instead of silicon alone in the oxide framework or by grafting organic groups along the walls.^39,45^ Fourth, it is imperative to ensure amine stability under humid cyclic conditions. Partially displacing wall-amine binding may compromise the stability of amines in the pores, which may eventually lead to amine loss. Lastly, the supported amines with less hydrophilic chains are candidates for humid DAC. Literature reports that poly(propylenimine), similar amine compounds to PEI but with propylene linkers between amine sites,^10^ exhibit less H_2_O uptake due to the less hydrophilic nature of longer aliphatic chains.

## Supporting Information Available

Basic characterization of PEI/SBA-15 composites, details of SANS and QENS theories and data analyses, and deuterated poly(ethylenimine) synthesis (PDF)
