# Supplementary material for: Probing the Distribution and Mobility of Aminopolymers after Multiple Sorption-Regeneration Cycles: Neutron Scattering Studies
Source: Ind Eng Chem Res. 2024 Aug 14;63(34):15100–12. doi: 10.1021/acs.iecr.4c01595 (PMC11363015; doi:10.1021/acs.iecr.4c01595)
Supplement: Supplementary file 1 — ie4c01595_si_001.pdf [file ie4c01595_si_001.pdf]

Supporting Information for

Probing the Distribution and Mobility of Aminopolymers after Multiple Sorption-Regeneration Cycles: Neutron Scattering Studies

Hyun June Moon,[1] William T. Heller,[2] Naresh C. Osti,[2] MinGyu Song,[1] Laura Proaño,[1] Ida Vaghefi,[1] and Christopher W. Jones[1*]

[1]School of Chemical & Biomolecular Engineering, Georgia Institute of Technology,

Atlanta, GA 30332, United States

[2]Neutron Scattering Division, Oak Ridge National Laboratory,

Oak Ridge, Tennessee 37831, United States

Email: cjones@chbe.gatech.edu

Table of Contents

1) Basic characterization of PEI/SBA-15 composites

2) SANS theory and data analysis

3) QENS theory and data analysis

4) Synthesis of deuterated poly(ethylenimine)

1) Basic characterization of PEI/SBA-15 composites

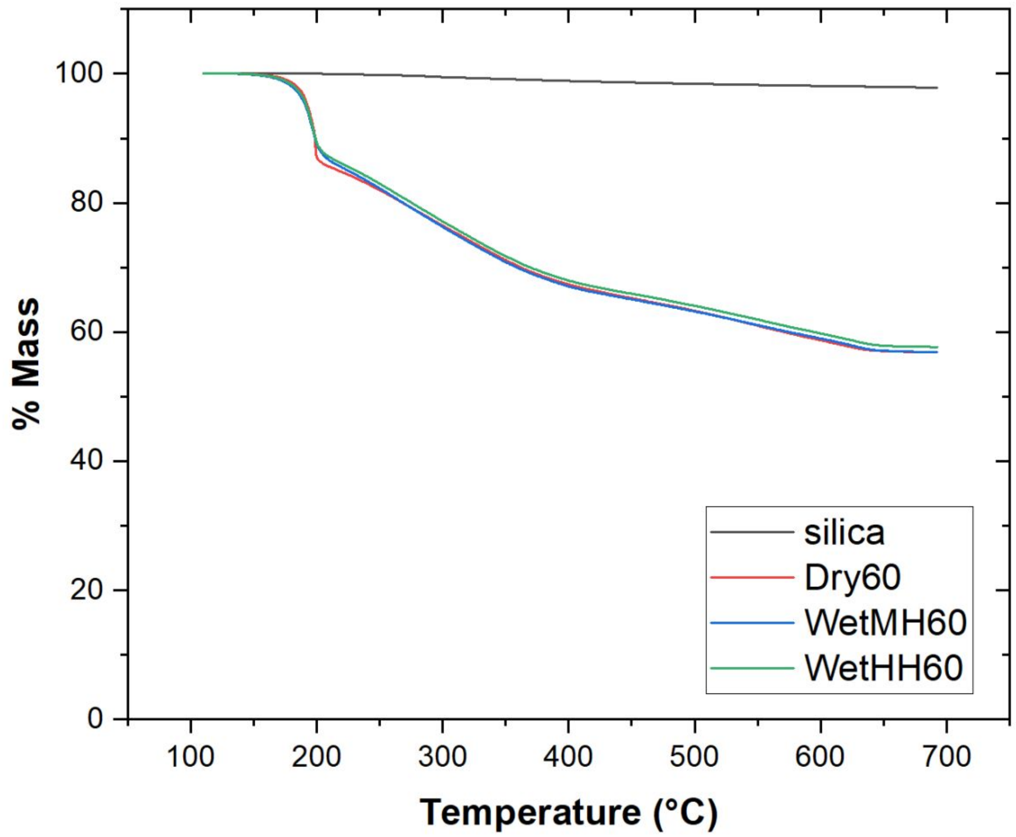

Figure S1. Combustion TGA results for post-cycled sorbents. Mass decrease from 120-700 °C suggests mass of PEI decomposed and removed from composite sorbents. Sorbents were pre-dried under 100 °C for 2 h under N_2 purge to remove adsorbed H_2O and CO_2.

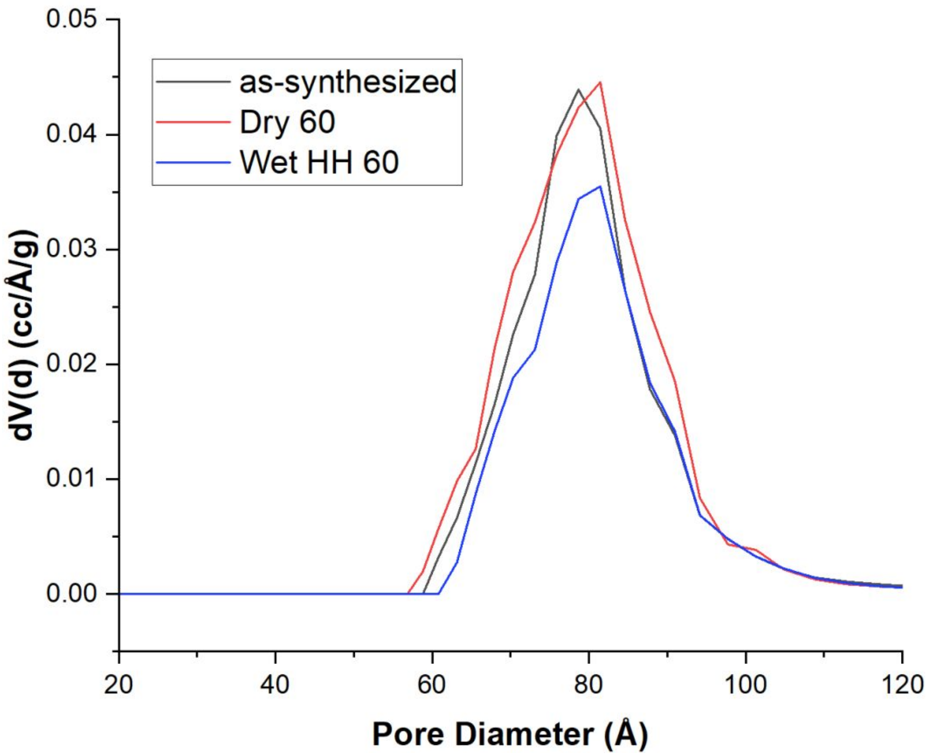

Figure S2. NLDFT pore size distribution determined by N_2 physisorption. All samples showed similar pore volumes and pore diameter, suggesting that there were no significant structural or macroscopic porosity differences due to different cyclic conditions.

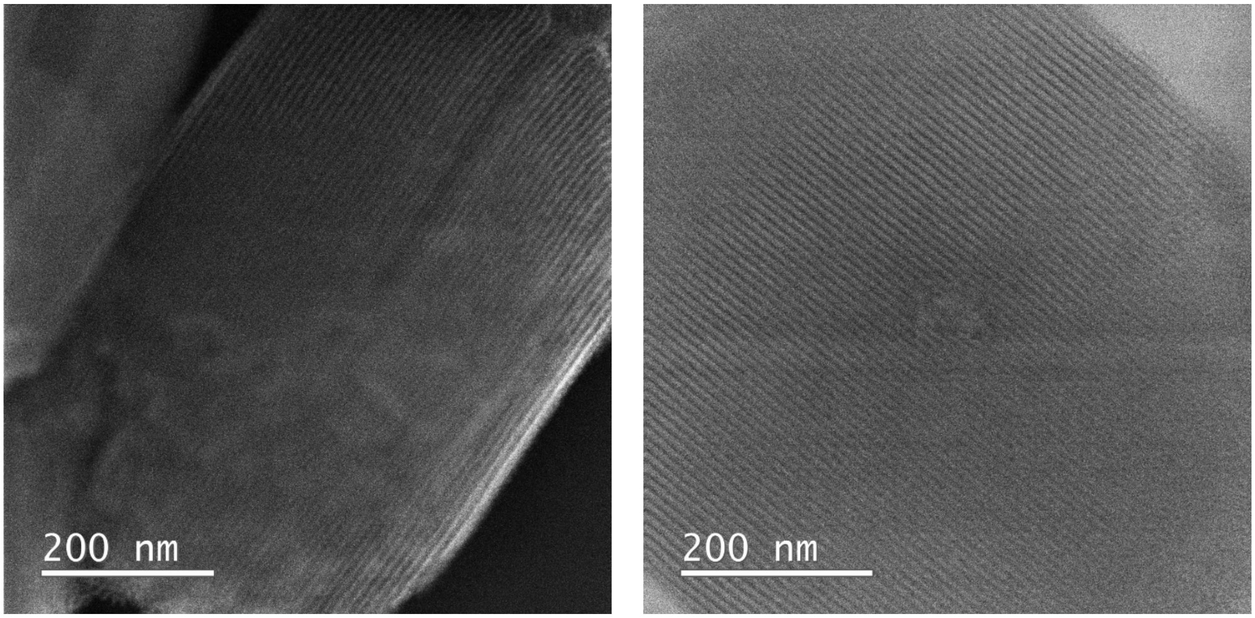

Figure S3. TEM images of SBA-15 silica used in this study.

2) SANS theory and data analysis

The SANS modeling and equation derivations were based on our previous SANS work.[1,2]

Model Derivation. The SBA-15 particles consist of numerous mesopores that can be treated as unit cells, C_i. The unit cells form hexagonal arrays as depicted in the right schematic. The scattered intensity to be obtained for the SBA-15 systems reflects the neutron SLD distribution. We start with the division of two regions, solid walls and voids. We can define the SLD within the i-th cell as $\rho_{C,i}$ and have the void space (evacuated) SLD as ρ_0. The scattered intensity can be expressed as equation S1.

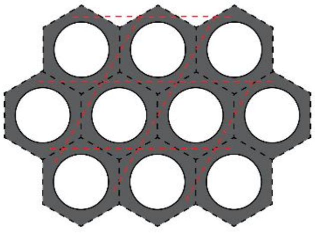

$$A(q) = \int_{V_{tot}} \rho(\boldsymbol{r}) e^{-i\boldsymbol{q}\cdot\boldsymbol{r}} d\boldsymbol{r} = \sum_{i}^{N_C} \int_{V_{C,i}} [\rho_{C,i}(\boldsymbol{r}_i) - \rho_0] e^{-i\boldsymbol{q}\cdot\boldsymbol{r}_i} d\boldsymbol{r}_i + \rho_0 \delta(\boldsymbol{q}) \qquad \text{(S1)}$$

Here $\boldsymbol{r}$ is the location vector, $\boldsymbol{q}$ is the momentum transfer vector of scattered neutron. To facilitate calculations, we can replace $\boldsymbol{r}_i$ for each unit cell with the sum $\boldsymbol{R}_i + \boldsymbol{u}$, where $\boldsymbol{R}_i$ is the vector for the center-of-mass of the reference unit cell. $\boldsymbol{u}$ is the displacement vector for unit cells. This treatment makes the volume integrals identical to within the phase factor $e^{i\boldsymbol{q}\cdot\boldsymbol{r}_i}$. With the positive magnitude of $\boldsymbol{q}$, the scattered amplitude can be converted to the scattered intensity $I(q) = A^*(q) \cdot A(q)$, and we arrive at equation S2.

$$I(\boldsymbol{q}) = \left\{ \iint_{V_C} [\rho_C - \rho_0](\boldsymbol{u}) [\rho_C - \rho_0](\boldsymbol{v}) e^{-i\boldsymbol{q}\cdot(\boldsymbol{u}-\boldsymbol{v})} d\boldsymbol{u} d\boldsymbol{v} \right\} \left\{ \sum_{i}^{N_C} \sum_{j}^{N_C} e^{i\boldsymbol{q}\cdot(\boldsymbol{R}_j - \boldsymbol{R}_i)} \right\} \qquad \text{(S2)}$$

The integrations over the unit cell volume yield a form factor, $P_C(\boldsymbol{q})$, which depicts a single mesopore domain. And the summation over phase factors yields a structure factor, $S(\boldsymbol{q})$, which illustrates arrangements of mesopore domains. Defining those terms leads to equation S3.

$$I(\boldsymbol{q}) = N_C P_C(\boldsymbol{q}) S(\boldsymbol{q}) \qquad \text{(S3)}$$

Here the unit cell form factor $P_C(\boldsymbol{q})$ is the squared magnitude of the Fourier transform (FT) of the unit cell SLD distribution such that $P_C(\boldsymbol{q})= F_C(\boldsymbol{q}) \cdot F_C^*(\boldsymbol{q})$ holds. To make calculations simpler, the coordinate can be re-defined. We can start with a rhombic primitive cell domain and transform into a rectangular domain as described below. This yields the widely known scattering law for a rectangular prism applicable for our calculations.[3]

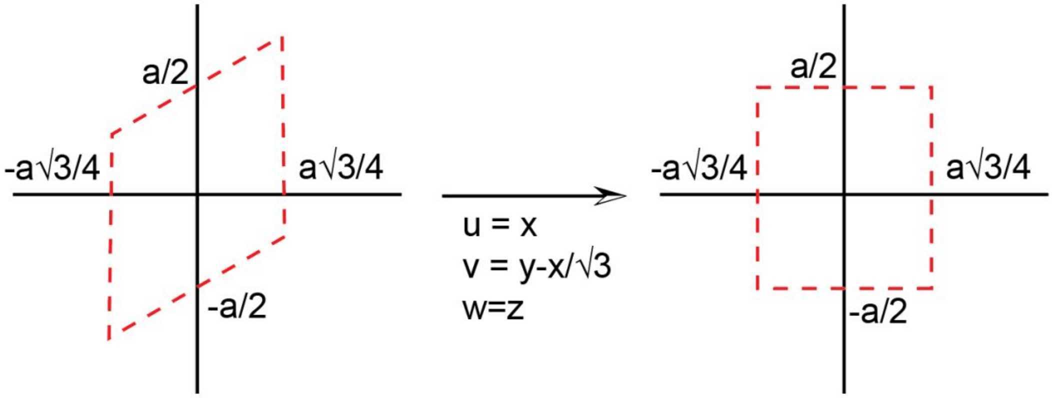

This treatment leads to equation S4 (below).

$$F(\boldsymbol{q}) = \frac{1}{V}\int_V e^{-i\boldsymbol{q}\cdot\boldsymbol{r}} d\boldsymbol{r} = \int_{-L/2}^{L/2} dw \cdot e^{-iq_z \cdot w} \int_{-a\sqrt{3}/4}^{a\sqrt{3}/4} du \cdot e^{-iq_x \cdot u} \int_{-a/2}^{a/2} dv \cdot e^{-iq_y \cdot \left(v+\frac{u}{\sqrt{3}}\right)} \quad \text{(S4)}$$

Integration leads to the following equation (equation S5)

$$F(\boldsymbol{q}) = \frac{\sin\,(q_z L/2)}{q_z L/2} \cdot \frac{\sin\,(q_y a/2)}{q_y a/2} \cdot \frac{\sin\,(q_x a\sqrt{3}/4 + q_y a/4\,)}{q_x a\sqrt{3}/4 + q_y a/4} \quad \text{(S5)}$$

Where L is the unit cell length and a is the characteristic length for a unit cell.

In constructing models for PEI distribution, the form factors for unit cells were constructed by the superposition principle. Incorporation of polymers in the pores brings about additional geometric contributors. For instance, polymer may adhere to the pore walls, or they may form aggregates. Those contributors were applied to the unit cell, which could be done by to adding or subtracting the corresponding amplitude, $F(\boldsymbol{q})$, dealing with specific shapes, weighted by the appropriate SLD and volume fractions. The pore region for polymer aggregate and pore-coating models were constructed from linear combinations of cylindrical scattering functions evaluated for radii and lengths determined by the polymer fill fraction. Dealing with the polymer aggregates in the pore centers, we can use the widely used cylindrical scattering functions, which can be expressed by equation S6.

$$F_{cylinder} = 2\,\frac{\sin\,(q_{axial} L/2)}{q_{axial} L/2} \cdot \frac{J_1\,(q_{radial} R)}{q_{radial} R} \quad \text{(S6)}$$

Where $J_1$is the Bessel function of the first kind. Plugging this to equation S5, we get equation S7.

$$F(\boldsymbol{q}) = F_{rhombus}V_{cell}(\rho_C - \rho_0) + F_{cylinder}(R_p, L_p)V_{pore}(\rho_0 - \rho_C) + F_{cylinder} - \rho_0) \tag{S7}$$

Where R_p is the radius of previously evacuated pore space (at which cylindrical polymer aggregates can locate), f is the polymer volumetric fill fraction, and ρ_{polym} is the polymer SLD.

The form factor, with powder scattering (with orientational/ensemble averaging), can be expressed as equation S8.

$$P(q) = \frac{2}{\pi}\int_0^{\pi/2}\int_0^{\pi/2} F^2(\boldsymbol{q}) \cdot \sin(\alpha) d\alpha d\beta \tag{S8}$$

The $\boldsymbol{q}$ vector can be decomposed for further calculations.
$q_x = q\sin(\alpha)\cos(\beta)$, $q_y = q\sin(\alpha)\sin(\beta)$, $q_z = q\cos(\alpha)$ – for rhombic axes
$q_{axial} = q\cos(\alpha)$, $q_{radial} = q\sin(\alpha)$ – for cylindrical axes

We next note that the ensemble average product $\langle P(\boldsymbol{q})S(\boldsymbol{q})\rangle$ is not equivalent to the product of the individual averages if the quantities are coupled. It can be shown that when the size and orientation of the scattering units are uncorrelated to position, the scattering of the ensemble can be well approximated as equation S9, following the reference.[4]

$$\left\langle \sum_i^{N_C}\sum_j^{N_C} \langle F_i(q)F_j^*(q)\rangle e^{i\boldsymbol{q}\cdot(\boldsymbol{R}_j - \boldsymbol{R}_i)} \right\rangle = N_C\left[\langle |F(\boldsymbol{q})|^2\rangle - |\langle F(\boldsymbol{q})\rangle|^2\right] - N_C|\langle F(\boldsymbol{q})\rangle|^2\langle S(\boldsymbol{q})\rangle \tag{S9}$$

In our case, the unit cells within the particle have fixed orientations, but the particles are randomly arranged, lacking correlations for distinct particles. Then we can reorder the ($i\,j$) indices of the phase factors (this leaves the amplitudes as originally indexed) while not changing the results. Rearranging the relation above permits the definition of a modified structure factor, where the ratio $|\langle F(\boldsymbol{q})\rangle|^2/\langle |F(\boldsymbol{q})|^2\rangle$ is defined as a cell-to-cell variance (later named as polydispersity). This treatment leads to a modified structure factor (as defined in equation S10).

$$S'(\boldsymbol{q}) = \left[1 + \frac{|\langle F(\boldsymbol{q})\rangle|^2}{\langle |F(\boldsymbol{q})|^2\rangle}\left(S(\boldsymbol{q})^{ideal} - 1\right)\right] \tag{S10}$$

We may then recast the relation $I(\boldsymbol{q}) \sim P(\boldsymbol{q})S'(\boldsymbol{q})$, approximating $|\langle F(\boldsymbol{q})\rangle|^2$ with the ideal, monodisperse form factor (those of the average) and derive $\langle |F(\boldsymbol{q})|^2\rangle$ with the polydispersity. Due to the heterogeneity of substructure in the unit cells (e.g., void, corona, polymer), we approximate the effect of polydispersity by convolution of the form factors with a linear combination of Gaussian and Lorentzian functions. By doing such, the spectral width was systematically varied to match the experimental data—i.e., matching the broadening beyond instrumental q-uncertainty, which was directly determined in the experimental data reduction routine. In this manner, an orientationally-averaged scattering law is given by $I(\boldsymbol{q}) \sim P(\boldsymbol{q})S'(\boldsymbol{q})$, using the ideal lattice structure factor as $S(\boldsymbol{q})^{ideal}$.

Our description of the ideal scattering system still needs to incorporate several more features of the real system. First, the collective interference of the unit cells, described by the structure factor, is manifested not only in the lattice positioning, but also in the hierarchical form of the particle that cells comprise. We thus expand the $S(\boldsymbol{q})^{ideal}$ as equation S11.

$$S(\boldsymbol{q}) = \left\{1 + \frac{1}{N_C}\sum_{i}^{N_C}\sum_{j\neq i}^{N_C} e^{i\boldsymbol{q}\cdot(\boldsymbol{R}_j - \boldsymbol{R}_i)}\right\} \tag{S11}$$

To account for the hierarchical particle form, we introduce a course-integration approximation, whereby we note that the summations approximate an integral over the region defined by the set of vectors $\{\boldsymbol{R_i}\}$ pointing to the unit cell centers of mass. If we multiply and divide the summation $\sum_i^{N_C} e^{i\boldsymbol{q}\cdot\boldsymbol{R}_i}$ by the volume increment $\Delta v = V_p/N_C$, we can approximate the summation as the normalized scattering amplitude of a homogeneous particle, leading to equation S12.

$$\frac{N_C}{V_p}\sum_{i}^{N_C} e^{i\boldsymbol{q}\cdot\boldsymbol{R}_i}\Delta v \sim \frac{N_C}{V_p}\int_{V_C} e^{-i\boldsymbol{q}\cdot\boldsymbol{r}} d\boldsymbol{r} = N_C \cdot F_{p0}(\boldsymbol{q}) \tag{S12}$$

With many unit cells ($N_C \gg 1$), the net form factor for the particle and the structure factor describing diffraction will dominate in different q domains. Therefore, we can approximate a total structure factor with the sum of the high and low q evaluations.[5] We set $N_C(N_C - 1) \sim {N_C}^2$ as $N_C \gg 1$, and revert to the orientational average to arrive at equation S13.

$$I(q) = N_C P_C(q)\{S'(q) + N_C P_{p0}(q)\} \tag{S13}$$

Here, the normalized bulk particle form factor $P_{p0}(q)$ will only be observed in surface scattering concerning large length scales, following the Porod's law. The current development is in units of cross section (m^2) per particle, making this term equate to $(S_p/{V_p}^2)q^{-4}$, where S_p is the exterior surface area of a particle and q^{-4} is the power law for the Porod's law.

The lattice structure factor is taken as that of 2D hexagonal lattice (equation S14). The lattice constant, a, can be determined from the peak positions observed in SANS spectra.

$$S(q) = 1 + \sum_{h,k}\delta(q_{hk}) \text{ where } q_{hk} = \frac{4\pi}{a\sqrt{3}}\sqrt{h^2 + k^2 + hk} \tag{S14}$$

To address q-dependent probability of diffracting plane orientation, the Lorentz factor $1/(2\sin(\theta))$ was applied. Then we permit for the possibility of 2-dimensional Debye-Waller factor $e^{-q^2\langle u^2\rangle/2}$, to account for deviation in the mean plane separations. By applying those, we arrive at the expanded structure factor as equation S15.

$$S(q) = \left[1 + \frac{1}{2\sin(\theta)} e^{-\frac{q^2\langle u^2\rangle}{2}} \sum_{h,k} \delta\left(\frac{4\pi}{a\sqrt{3}}\sqrt{h^2 + k^2 + hk}\right)\right] \tag{S15}$$

Lastly, the scattering at medium-high q appeared in the observed SANS spectra showed tendency of a smooth decay. This can be attributed to small-scale structural inhomogeneities such as micropores (or intra-wall pores) and surface roughness, as well as additional factors from occlusions and crystalline domains generating density fluctuations within solid domains (i.e., condensed SiO_2 phase). In our previous article,[1] we approximated this scattering contribution to a random, isotropic two-phase continuous medium (as described by Debye et al),[6,7] which succeed to capture microscopic structural clues for PEI around walls. Such a medium scatters with a squared Lorentzian intensity distribution, defined by an effective correlation length, ξ (i.e., characteristic length we observe structural inhomogeneities). The intensity is proportional to the mean squared density fluctuations $\langle\eta^2\rangle = \langle\rho^2\rangle - \langle q\rangle^2$ (i.e., the extent of SLD fluctuation within solid domains) and the illuminated volume, which we presently define as the solid volume per particle. This arrives at equation S16.

$$i_{diff}(q) = \frac{8\pi\xi^3\langle\eta^2\rangle V_s}{(1 + q^2\xi^2)^2} \quad \text{(S16)}$$

We underline that entry of the polymer chains into a corona layer may bring about additional contributors, possibly anisotropic behaviors. Those anisotropic trends could be tackled by using two Lorentzian functions. However, given the small volume fraction and small length scales, together with powder averaging and numerous contributors to diffuse scatter, we could not capture noticeable improvements with two Lorentzian functions, and we consider that small-scale factors were not discernible from the current data.

Combining the unit cell scattering with the structure factors defined previously, we arrive at a final scattering law, as shown in equation S17.

$$I(q) = \frac{N_p}{V}\left[P_c \cdot \left\{N_c S(q) + {N_c}^2 \cdot \frac{S_p}{{V_p}^2} q^{-4}\right\} + \frac{8\pi\xi^3\langle\eta^2\rangle V_s}{(1 + q^2\xi^2)^2}\right] \quad \text{(S17)}$$

The description of the parameters can be found below.

N_p: number of particles in a sample cell
V: volume of sample cell
P_c: form factor of mesopore ($P_c(\boldsymbol{q}) = F_c(\boldsymbol{q}) \cdot F_c^*(\boldsymbol{q})$)
$S(q)$: structure factor
N_c: number of unit cells
S_p: external surface area of a silica particle
ξ: correlation length
$\langle\eta^2\rangle$: SLD fluctuation ($\langle\eta^2\rangle = \langle\rho^2\rangle - \langle\rho\rangle^2$)
V_s: solid skeletal volume (i.e., volume of nonporous region of a silica particle)

SANS Data Fitting Routine. To assess structural properties of the evacuated SBA-15 silica and PEI/SBA-15 composites, the observed SANS spectra were fitted against the aforementioned theoretical models. A library of models was built covering a broad range of structural parameters. Some parameters were pre-defined a priori with suitable experimental observations (e.g., N_2

physisorption for polymer fill fraction, lattice constant from Bragg peak [10] positions). To quantitatively analyze PEI distribution, grids of the structural parameters including pore radius, corona layer thickness, and corona layer SLD values were defined. For any parameters accounting for lengths, a step of 1 Å was applied. For corona SLD values, we set a basis SLD of 3.5×10^{-6} Å^{-2} (SiO_2) (as reported in the literature)[1,8] and then defined a range of multipliers (0.01 - 3.0, with logarithmically spaced steps).

In accounting for the relatively macroscopic distribution of PEI (i.e., how much PEI molecules are deposited on the walls), models with distinguishable polymer distributions were created. Representative cases are 1) continuous deposition of polymers from the pore walls (core-shell), 2) consistent buildup of polymer aggregates at pore centers (plug), and 3) mixed cases including the polymer deposition (occupying a certain extent of pore volume) and subsequent formation of polymer plugs (sequential). We bear in mind that the complex nature of polymer/silica composites and the nature of low MW PEI (~800 g/mol) may not lead to samples that fall into such distinctive categories. However, considering the synthetic protocols—where the slurry of polymer and silica (in MeOH) was gradually evaporated to allow for polymer entry to the pores—and the chemical identities of polymeric amines or polymeric alcohols and silanols (on pore surfaces), we would expect a more solid-like polymeric regime around pore walls against more liquid-like polymers (which are likely similar to free, non-confined entities).

Lastly, the scattering law stated earlier in this section contains several variables that may not be uniquely determinable. We chose to evaluate the parameters that could be safely estimated to arrive at suitable values. Specifically, the term $N_p N_c / V$ can be reduced to the ratio of the particle packing fraction and unit cell volume (φ / V_c). Utilizing the known lattice parameter, an estimated packing fraction of 0.6 (typical of powders), and a pore length of 1 μm (based on particle size seen in TEM images, **Figure S3**), terms such as $\langle \eta^2 \rangle$ and S_p / V_p can be extracted from a curve fit. No attempt was made to fit absolute diffraction intensities; rather, a magnitude was determined from the first peak and S(q) scaled to this value.

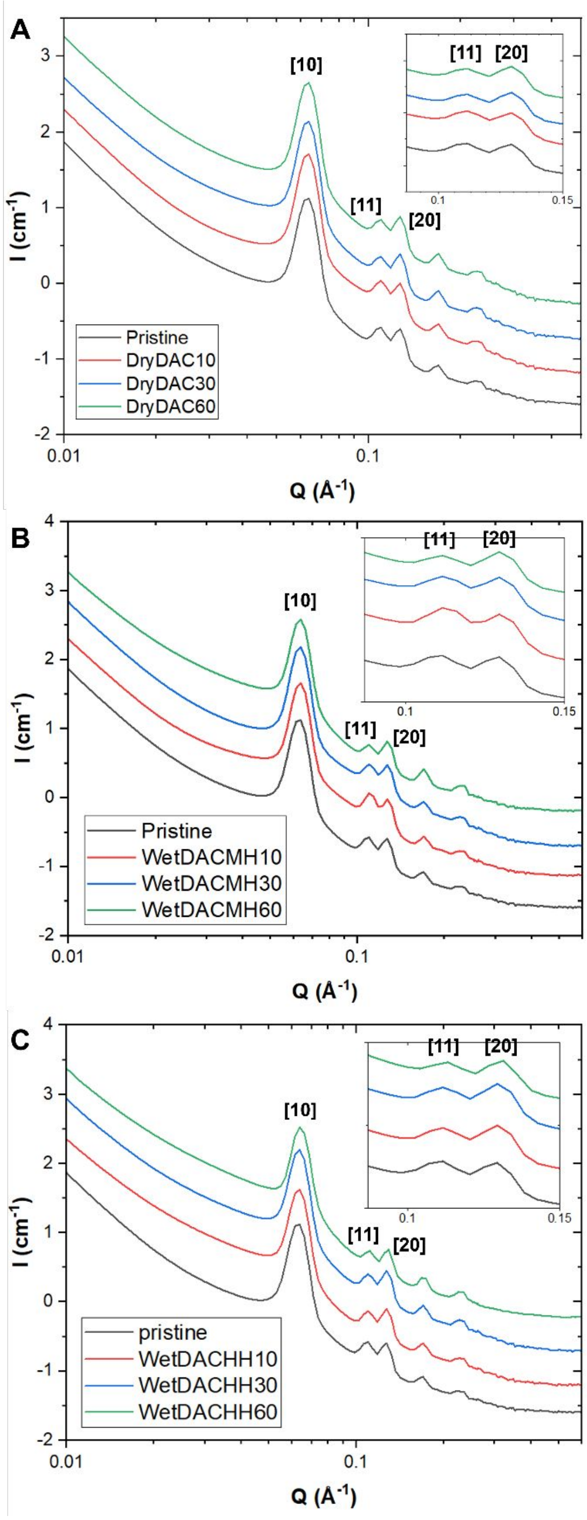

Figure S4. SANS spectra for all samples investigated, with key diffraction peaks ([10], [11], [20]) highlighted. SANS curve fit results are summarized in **Table S1**.

Table S1. Structural parameters extracted from SANS curve fit. Highlighted cells include meaningful deviation from the fresh, as-synthesized sorbent.

	Model	R_p (Å)	t_c (Å)	corona SLD (10^{-6} Å^{-2})	Corr. length (ξ) (Å)	SLD fluct. $\langle\eta\rangle^2$(Å^{-4})
Evacuated SBA-15	-	38	10	0.35	7	2.64
As-synthesized	20 vol% shell	34	12	0.8	11	1.45
Dry 10	20 vol% shell	34	14	0.9	10	1.40
Dry 30	10 vol% shell	36	6	0.8	11	1.56
Dry 60	10 vol% shell	36	6	0.8	10	1.66
Wet MH 10	20 vol% shell	34	16	1.0	12	1.32
Wet MH 30	20 vol% shell	34	8	0.8	10	1.08
Wet MH 60	20 vol% shell	34	14	0.5	13	1.10
Wet HH 10	20 vol% shell	36	8	0.2	11	1.26
Wet HH 30	20 vol% shell	36	10	0.2	11	1.12
Wet HH 60	20 vol% shell	35	12	0.15	14	1.10

We gather a sense of the microscopic structures penetrating the pore walls by considering the diffuse scattering. In our SANS model, there are two parameters in diffuse scattering—one is the correlation length (ξ) and another is the SLD fluctuation ($\langle\eta\rangle^2 = \langle\rho^2\rangle - \langle\rho\rangle^2$), whose values are denoted in **Table S1**. The correlation length (ξ) gauges the length scale where we observe fluctuation of SLD values. SLD fluctuation in the table above represents the extent of SLD fluctuation given the population of the scattering contributors. For blank SBA-15, the non-zero correlation length and SLD fluctuation indicate structural defects through the pore walls, such as rough surfaces, micropores, and occluded pores, as mentioned earlier. The SLD fluctuation could be attributed to the contrast term between condensed silica and void spaces. In as-synthesized PEI/SBA-15 we have larger correlation length but reduced SLD fluctuation. This suggests a disturbance of wall-void interfaces due to contributors such as PEI and H_2O. Now comparing cycled samples against the fresh sorbent, dry conditions led to comparable values while wet cycles led to increased correlation lengths and reduced SLD fluctuation. We consider that hydrated PEI chains could partly penetrate the micropores, augmenting correlation lengths but mitigating SLD fluctuation given by the inclusion of this positive SLD contributor.

3) QENS theory and data analysis

The QENS technique measures correlations between the initial position of a scatterer and its position later in time via the measurement of the energy and momentum transfer between the scatterer and incident neutrons. This self-correlation can be interpreted from the incoherent scattering law, S(**Q**,ω), which is a Fourier-transform of the self-correlation function, G_s(**r**,t), of the general Van Hove correlation function (equation S18).[9]

$$S(\boldsymbol{Q},\omega) = \iint G_s(\boldsymbol{r},t)\, e^{i(\omega t - \boldsymbol{Q}\cdot\boldsymbol{r})} d\boldsymbol{r} dt \qquad \text{(S18)}$$

As diffusive motions exhibit exponential decay in their correlation functions and given that the exponentials translate to Lorentzian functions in the frequency space, QENS spectra resulting from diffusive processes can be expressed as a linear combination of Lorentzian functions, $L_{\mathrm{j}}(Q,\omega)$ and the relationship below (equation S19), with definition of the Lorentzian function (equation S20).

$$S(Q,\omega) = \left[1 - \sum A_{\mathrm{j}}(Q)\right] \cdot \delta(Q) + \sum A_{\mathrm{j}}(Q) \cdot L_{\mathrm{j}}(Q,\omega) \tag{S19}$$

$$L_{\mathrm{j}}(Q,\omega) = \frac{1}{\pi} \frac{\Gamma_{\mathrm{j}}(Q)}{\Gamma_{\mathrm{j}}^{2}(Q) + (\hbar\omega)^{2}} \tag{S20}$$

The above powder-averaged scattering law is applicable for our samples, with the magnitude of the momentum transfer vector Q and $A_{\mathrm{j}}(Q)$ being the spectral weights for each component function. For confined processes, the spectral weights, $A_{\mathrm{j}}(Q)$ can be related to the geometry of the confinement space (which later will be used for EISF calculation).[8] The functionality of the Lorentzian half-width at half-maximum (HWHM) is denoted as $\Gamma_{\mathrm{j}}(Q)$, which encodes characteristic time and length scales of the corresponding motion. Specific models for the spectral weights and Lorentzian widths are discussed further below in the context of the data. Instrumental resolution, $R(Q,\omega)$, and a flat background function, $B(Q)$, were incorporated as shown in equation S21:

$$I(Q,\omega) = [S(Q,\omega) + B(Q)] \otimes R(Q,\omega) \tag{S21}$$

All model fitting was performed by minimizing residuals. Each Γ_{j} value was sampled by a full-factorial parameter sweep over a predetermined range, while all coefficients (spectral widths and backgrounds) were determined at each set of Γ_{j}'s by weighted multiple linear regression.

Lastly, for EISF fits, we used two Lorentzian functions, a delta function (i.e., QENS resolution), and a flat background to capture the QENS spectrum. The effective QENS contribution from hPEI can be represented by two Lorentzian broadenings, whose spectral weights could be determined by the curve fitting routines. Those spectral weights, in turn, can be used to understand the extent of PEI mobility concerning each Lorentzian. Assuming there are some fractions, c, of immobile scattering and two dynamic processes reflected by Lorentzian functions, we can arrive at the scattering laws for those two separate processes, following equation S22 (for slow, global motions) and equation S23 (for fast, local motions).

$$S_1(Q,\omega) = c_1\delta(Q) + (1 - c_1)L_1(Q,\omega) \tag{S22}$$

$$S_2(Q,\omega) = c_2\delta(Q) + (1 - c_2)\{[1 - A_2(Q)]\delta(Q) + A_2(Q)L_2(Q,\omega)\} \tag{S23}$$

Here we have separate fractions of immobility (c_1 and c_2 for slower (global) and faster (local), respectively), $L_1(Q,\omega)$, where $L_2(Q,\omega)$ denotes Lorentzian functions associated with slower (global) and faster (local) motions, respectively, and $A_2(Q)$ is the spectral weight for the second Lorentzian contributor. Based on equations S22 and S23, the overall scattering law can be expressed as the convolution of those two scattering contributions (as shown in equation S24).

$$S(Q,\omega) = S_1(Q,\omega) \otimes S_2(Q,\omega) \tag{S24}$$

This can be expanded to equation S25.

$$\begin{gathered} S(Q,\omega) = \{c_1c_2 + [1 - A_2(Q)]c_1(1 - c_2)\}\delta(Q) \\ + \{(1 - c_1)c_2 + (1 - c_1)(1 - c_2)[1 - A_2(Q)]\}L_1(Q,\omega) \\ + \{c_1(1 - c_2)A_2(Q)\}L_2(Q,\omega) + \{(1 - c_1)(1 - c_2)A_2(Q)\}L_1(Q,\omega) \otimes L_2(Q,\omega) \end{gathered} \tag{S25}$$

Here L_1 is much narrower than L_2, therefore L_1 can be treated as a delta function in respect to L_2 in their convolution ($L_1(Q,\omega) \otimes L_2(Q,\omega) \sim L_2(Q,\omega)$). This leads to equation S26.

$$\begin{gathered} S(Q,\omega) = \{c_1c_2 + [1 - A_2(Q)]c_1(1 - c_2)\}\delta(Q) \\ + \{(1 - c_1)c_2 + (1 - c_1)(1 - c_2)[1 - A_2(Q)]\}L_1(Q,\omega) \\ +(1 - c_2)A_2(Q)L_2(Q,\omega) \end{gathered} \tag{S26}$$

Since the fractional contributions sum to unity, the EISF from equation S26 can be simplified as equation S27.

$$\text{EISF} = c_1c_2 + [1 - A_2(Q)]c_1(1 - c_2) \tag{S27}$$

Therefore, the elastic component appears as a weighted sum between a constant representing the totally immobile fraction of scatterers and a term proportional to the isolated EISF of the fast process. In the event that L_2 broadening is too large to be detected in the instrument energy window, the quasielastic neutrons are not detected, while the elastic fraction still contributes to measured signal and increases the apparent EISF.

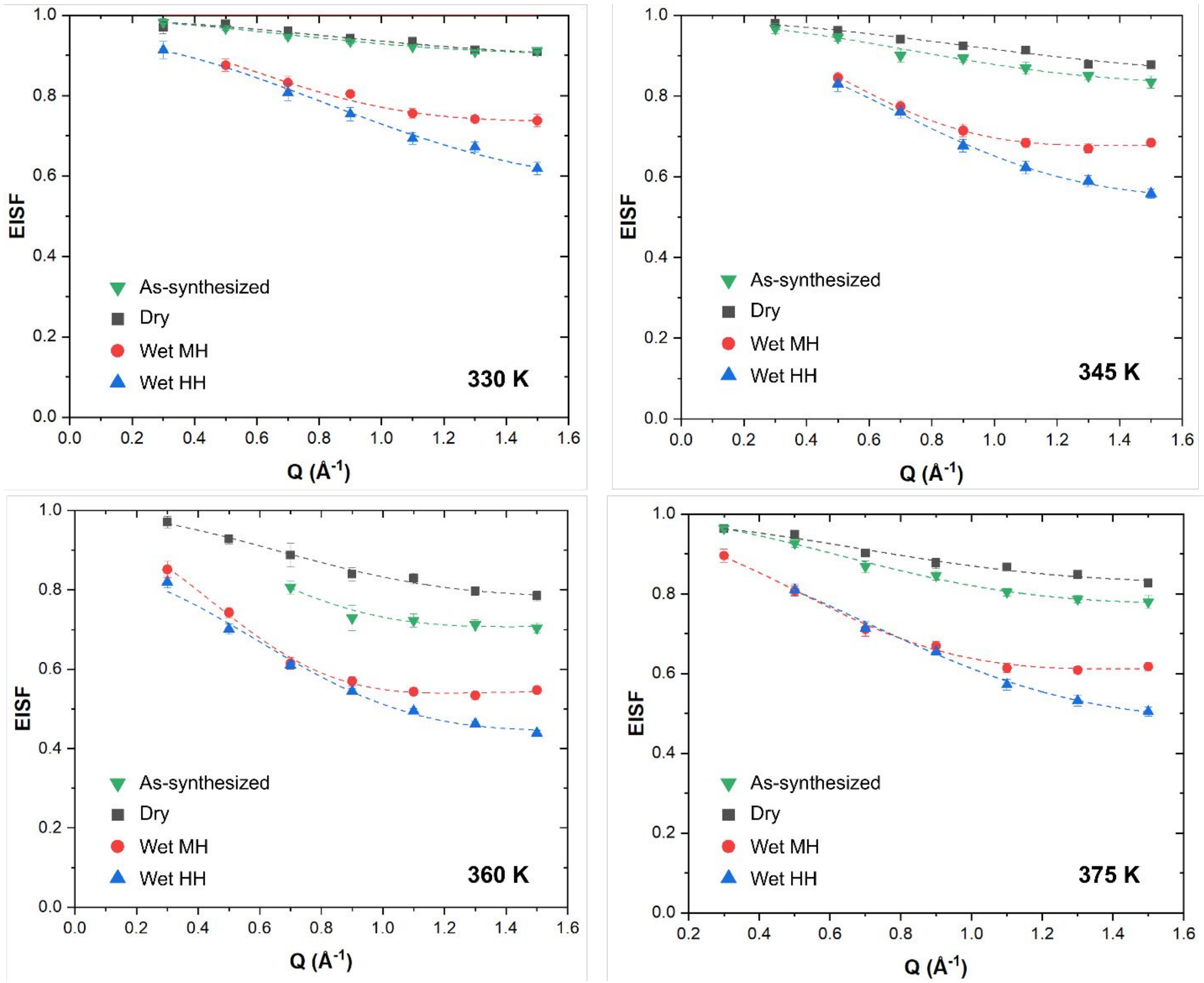

Figure S5. EISF plots at varied temperatures (330, 345, 360, 375 K). Dotted lines denote curve fits against theoretical model (equation 3, derivation can be found earlier in this section). Extracted dynamic parameters are denoted in **Tables S2-4**.

Table S2. EISF dynamic parameters at 330 K. R_0 denotes confinement length, c_1 and c_2 stand for extent of immobile PEI regards with slower (global) and faster (local) diffusive motions, respectively. R^2 values suggest that fits were reliable.

T= 330 K	Pristine	Dry 60	WetMH 60	WetHH 60
R_0	2.37	2.02	2.86	2.12
c_1	0.98	0.98	0.96	0.94
c_2	0.91	0.91	0.77	0.64
R^2	0.999	0.999	0.999	0.980

Table S3. EISF dynamic parameters at 345 K.

T= 345 K	Pristine	Dry 60	WetMH 60	WetHH 60
R_0	2.29	1.88	3.32	2.45
c_1	0.98	0.98	0.98	0.93
c_2	0.85	0.87	0.69	0.59
R^2	0.979	0.959	0.988	0.996

Table S4. EISF dynamic parameters at 375 K.

T= 375 K	Pristine	Dry 60	WetMH 60	WetHH 60
R_0	3.19	2.54	3.72	2.81
c_1	0.99	0.99	0.95	0.85
c_2	0.71	0.79	0.57	0.53
R^2	0.953	0.989	0.992	0.986

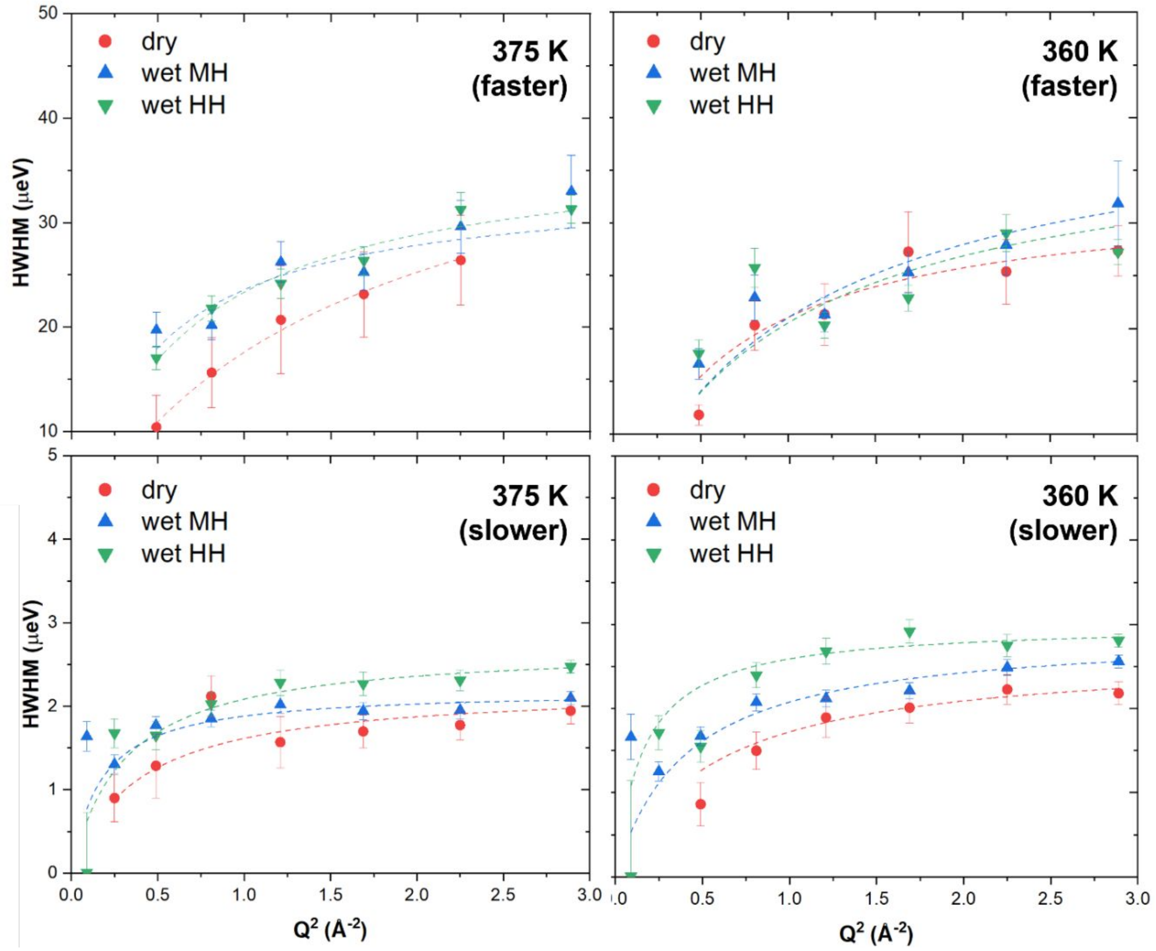

Figure S6. HWHM plots for slower and faster diffusive PEI motions. The dotted lines denote HWHM fits against the theoretical model (equation 5; $\Gamma_i(Q) = \frac{\hbar}{\tau}\left(1 - \frac{1}{1+DQ^2\tau}\right)$).

Table S5. HWHM fit results for slow, center-of-mass diffusion of PEI at 375 K. D, τ, and $\langle L \rangle^{21/2}$ denote diffusivity, timescale for jump motions, and square root of the mean-squared average jump lengths, respectively.

T= 375 K (slow)	Dry 60	WetMH 60	WetHH 60
D ($\times 10^{-10}$ m^2/s)	0.89	2.00	1.36
τ (ns)	0.29	0.30	0.24
$\langle L \rangle^{21/2}$ (Å)	3.96	6.00	4.45

Table S6. HWHM fit results for slow, center-of-mass diffusion of PEI at 360 K. D, τ, and $\langle L \rangle^{21/2}$ denote diffusivity, timescale for jump motions, and square root of the mean-squared average jump lengths, respectively.

T= 360 K (slow)	Dry 60	WetMH 60	WetHH 60
D ($\times 10^{-10}$ m^2/s)	0.74	1.10	2.85
τ (ns)	0.25	0.23	0.21
$\langle L \rangle^{21/2}$ (Å)	3.32	3.85	6.12

4) dPEI synthesis and characterization

Materials. Ethanol-1,1,2,2-d_4-amine (CDN Isotopes), ethylene-d_4-diamine·2HCl (CDN Isotopes), hydrobromic acid (48%, Sigma-Aldrich), ethyl ether (Sigma-Aldrich), and sodium hydroxide (Sigma-Aldrich) were used for dPEI synthesis. Details of the synthesis are described below.

Overview. dPEI was synthesized by the acid-catalyzed ring-opening polymerization of ethylenimine-d_4 (aziridine-d_4) with 2HCl-coupled ethylenediamine-d_4 as an initiator as well as a capping agent. The ethylenimine-d_4 was synthesized first via the bromination of ethanol-amine-d_4 (CDN isotopes) to bromoethylamine-d_4 by heating in HBr. Secondly, base-activated ring closure was applied to bromoethylamine-d_4 to form ethylenimine-d_4, after which vacuum distillation was carried out to concentrate the ethylenimine-d_4. Lastly, acid-catalyzed ring-opening polymerization was adopted for polymerization of ethylenimine-d_4, which yielded deuterated PEI (dPEI).

Synthesis of 2-bromoethyl-d_4-amine·HBr. We followed the procedure described in our previous article.[1] 0.1 mol of ethanol-1,1,2,2-d_4-diamine was added dropwise to 50 mL 48% HBr (0.44 mol) in a round-bottom flask while stirring in an ice bath. The distillation head was attached (making sure there was no leak on the junctions), another round-bottom flask was connected as a receiver. The reaction flask was heated to 145 °C while the receiver flask was placed in an ice bath. After 8 hours, the reaction system was cooled to ~30 °C, and additional 25 mL HBr was added, after which the reaction proceeded for additional 6 hours at 145 °C. After the reaction, the flask was cooled to ~30 °C, and a rotary evaporator was used to remove H_2O and residual HBr from the flask, after which crystallization occurred and brown solid fraction was obtained. The brown solid fraction was rigorously rinsed with ethyl ether to remove molecular bromine, until pearlescent white precipitation was obtained.

Synthesis of ethylenimine-d_4 (aziridine-d_4). **[Hazard note: ethylenimine (aziridine) is highly volatile, toxic and is a strong irritant to the respiratory systems and skin. This is an alkylating agent that may induce mutation of DNA and is classified as a possible carcinogen. Use with extra caution, handle this in a fume hood, where proper personal protective equipment, and**

keep isolated aziridine sufficiently chilled (recommended: dry ice and acetone temperatures). Wash any items potentially contaminated with aziridine using mild acids such as acetic acid.] The synthetic protocol was adapted from our previous article.[1] First, 20 g of the 90% 2-bromoethyl-d_4-amine were dissolved in 50 mL deionized water and added to an NaOH (aq) prepared from 34 mL 50 wt% NaOH (aq) and 16 mL deionized water. A distillation head with a vacuum port and a cooling jacket was connected to the reaction flask, and ice water flowed through the cooling jacket to maintain temperature of ~0 °C. The reaction flask was initially heated to 40 °C for 30 min, ramped to 65 °C in ~10 °C/hour ramp rate, after which the static vacuum was applied until gentle bubbling was observed. The bottom of the receiver flask was in contact with a liquid nitrogen bath to freeze distilled aziridine. After ~6 hours of vacuum distillation, approximately 10 mL of distillate was collected, which was transferred to a 50 mL sealable plastic centrifuge tube. To remove H_2O, the distilled phase was salted with NaOH pellets, during which the tube was swirled in a cooling bath (dry ice and acetone) to prevent overheating. After adding ~7 g of NaOH, a phase separation was observed, where the bottom phase was NaOH salted water and the top phase was aziridine. The top part was pipetted off, collected to a sealable tube, and then the volume and mass was measured to estimate the density.

Synthesis of deuterated PEI. Acid-catalyzed ring-opening polymerization was deployed, following the procedures found in literature and our previous article.[1,10,11] First, a solution of 0.3 g ethylene-d_4-diamine·2HCl (capping agent and acid catalyst) was prepared in 3 mL of deionized water. 2 mL of 90 wt% aziridine-d_4 was added to the aqueous solution of ethylene-d_4-diamine·2HCl in 0.5 mL aliquots at 30-minute intervals to maintain moderate polymerization rate and control heat generated during ring opening reactions. The estimated monomer : capping agent ratio was 17:1. After 4 hours, 10 mL water was added with 25 mL Ambersep 900 (base) resin to remove chloride. The suspension was rigorously filtered using syringe filter (PTFE, 0.45 µm), and rotary evaporator was used to remove water.

Characterization. ^{2}H NMR spectra were taken (using Bruker Avance III 400 MHz) to estimate the purity of 2-bromoethyl-d_4-amine and the chemistries of ethylenimine-d_4 and deuterated poly(ethylenimine) (**Figure S7**). A quantitative, inverse-gated ^{13}C NMR spectrum was taken using the same instrument (Bruker Avance III 400 MHz) (**Figure S8**). Aqueous gel permeation chromatography reported an estimated MW of ~850 g/mol, slightly smaller number of atoms compared to commercial PEI.

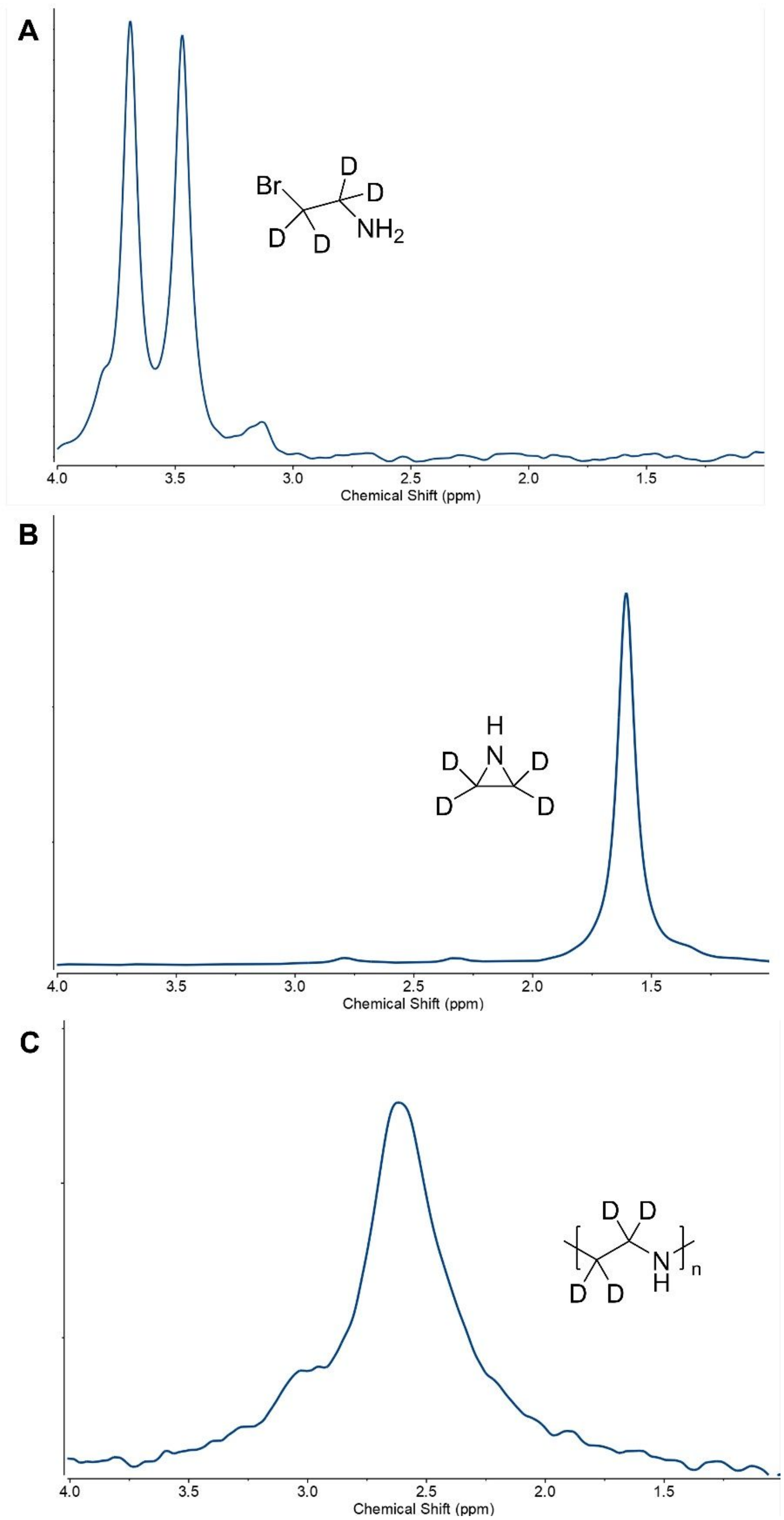

Figure S7. ^{2}H NMR spectra for (A) 2-bromoethyl-d$_4$-amine, (B) aziridine-d$_4$, and (C) deuterated PEI. Estimated purity of 2-bromoethyl-d$_4$-amine was approximately 90% (balance ~10% unreacted ethanol-1,1,2,2-d$_4$-amine). Estimated purity of aziridine-d$_4$ was approximately 90 wt% with balance water (calculated based on mass density, following the literature).[12]

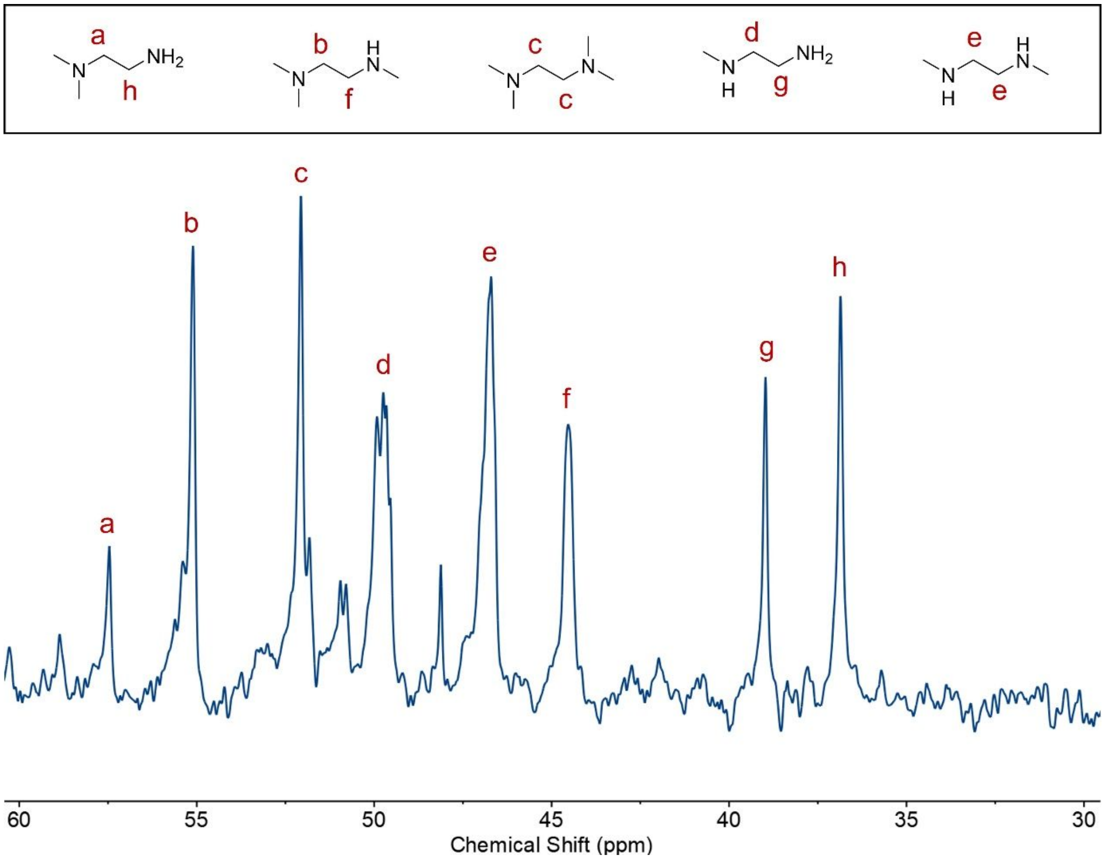

Figure S8. ^{13}C NMR spectrum and quantification (solvent: D_2O). Ratios of amines based on the integrations are 29 % primary amines, 46 % secondary amines, and 25 % tertiary amines. The ratios of primary, secondary, and tertiary amines were determined (42%, 38%, 20%, respectively), comparable to the commercial branched PEI (44%, 33%, 23%).[13]

References

(1) Holewinski, A.; Sakwa-Novak, M. A.; Jones, C. W. Linking CO2 Sorption Performance to Polymer Morphology in Aminopolymer/Silica Composites through Neutron Scattering. *Journal of the American Chemical Society* **2015**, *137* (36), 11749–11759.

(2) Moon, H. J.; Carrillo, J.-M. Y.; Song, M.; Rim, G.; Heller, W. T.; Leisen, J.; Short, G. N.; Banerjee, S.; Sumpter, B. G.; Jones, C. W. Underlying Roles of Polyol Additives in Promoting CO2 Capture in PEI/Silica Adsorbents. *Submitted.*

(3) Pedersen, J. S. Analysis of Small-Angle Scattering Data from Colloids and Polymer Solutions: Modeling and Least-Squares Fitting. *Advances in colloid and interface science* **1997**, *70*, 171–210.

(4) Kotlarchyk, M.; Chen, S.-H. Analysis of Small Angle Neutron Scattering Spectra from Polydisperse Interacting Colloids. *The Journal of chemical physics* **1983**, *79* (5), 2461–2469.

(5) Spalla, O.; Lyonnard, S.; Testard, F. Analysis of the Small-Angle Intensity Scattered by a Porous and Granular Medium. *Journal of applied crystallography* **2003**, *36* (2), 338–347.

(6) Debye, P. Bueche, AM. *J. Appl. Phys.* **1949**, *1949*, 20–518.

(7) Debye, P.; Anderson Jr, H.; Brumberger, H. Scattering by an Inhomogeneous Solid. II. The Correlation Function and Its Application. *Journal of applied Physics* **1957**, *28* (6), 679–683.

(8) Chiang, W.-S.; Fratini, E.; Baglioni, P.; Georgi, D.; Chen, J.-H.; Liu, Y. Methane Adsorption in Model Mesoporous Material, SBA-15, Studied by Small-Angle Neutron Scattering. *The Journal of Physical Chemistry C* **2016**, *120* (8), 4354–4363.

(9) Van Hove, L. Correlations in Space and Time and Born Approximation Scattering in Systems of Interacting Particles. *Physical Review* **1954**, *95* (1), 249.

(10) Jones, G. D.; Langsjoen, A.; NEUMANN, S. M. M. C.; Zomlefer, J. The Polymerization of Ethylenimine. *The Journal of Organic Chemistry* **1944**, *9* (2), 125–147.

(11) Zhuk, D. S.; Gembitskii, P. A.; Kargin, V. A. Advances in the Chemistry of Polyethyleneimine (Polyaziridine). *Russian Chemical Reviews* **1965**, *34* (7), 515.

(12) Pascoe, P.; Sherbrogk-Gox, W. The Reaction between Anhydrous Ethyleneimine and Water. *Journal of Applied Chemistry* **1963**, *13* (12), 564–572.

(13) Drese, J. H.; Choi, S.; Lively, R. P.; Koros, W. J.; Fauth, D. J.; Gray, M. L.; Jones, C. W. Synthesis–Structure–Property Relationships for Hyperbranched Aminosilica CO2 Adsorbents. *Advanced Functional Materials* **2009**, *19* (23), 3821–3832.
